# Unraveling the Genetic Basis of Seed Tocopherol Content and Composition in Rapeseed (*Brassica napus *L.)

**DOI:** 10.1371/journal.pone.0050038

**Published:** 2012-11-20

**Authors:** Xingxing Wang, Chunyu Zhang, Lingjuan Li, Steffi Fritsche, Jessica Endrigkeit, Wenying Zhang, Yan Long, Christian Jung, Jinling Meng

**Affiliations:** 1 National Key Laboratory of Crop Genetic Improvement, Huazhong Agricultural University, Wuhan, China; 2 Plant Breeding Institute, Christian-Albrechts-University, Kiel, Germany; 3 Institute of Plant Genetics and Breeding, School of Agriculture, Yangtze University, Jingzhou, China; Kansas State University, United States of America

## Abstract

**Background:**

Tocopherols are important antioxidants in vegetable oils; when present as vitamin E, tocopherols are an essential nutrient for humans and livestock. Rapeseed (*Brassica napus* L, AACC, 2 n = 38) is one of the most important oil crops and a major source of tocopherols. Although the tocopherol biosynthetic pathway has been well elucidated in the model photosynthetic organisms *Arabidopsis thaliana* and *Synechocystis* sp. PCC6803, knowledge about the genetic basis of tocopherol biosynthesis in seeds of rapeseed is scant. This project was carried out to dissect the genetic basis of seed tocopherol content and composition in rapeseed through quantitative trait loci (QTL) detection, genome-wide association analysis, and homologous gene mapping.

**Methodology/Principal Findings:**

We used a segregating Tapidor × Ningyou7 doubled haploid (TNDH) population, its reconstructed F_2_ (RC-F_2_) population, and a panel of 142 rapeseed accessions (association panel). Genetic effects mainly contributed to phenotypic variations in tocopherol content and composition; environmental effects were also identified. Thirty-three unique QTL were detected for tocopherol content and composition in TNDH and RC-F_2_ populations. Of these, seven QTL co-localized with candidate sequences associated with tocopherol biosynthesis through *in silico* and linkage mapping. Several near-isogenic lines carrying introgressions from the parent with higher tocopherol content showed highly increased tocopherol content compared with the recurrent parent. Genome-wide association analysis was performed with 142 *B. napus* accessions. Sixty-one loci were significantly associated with tocopherol content and composition, 11 of which were localized within the confidence intervals of tocopherol QTL.

**Conclusions/Significance:**

This joint QTL, candidate gene, and association mapping study sheds light on the genetic basis of seed tocopherol biosynthesis in rapeseed. The sequences presented here may be used for marker-assisted selection of oilseed rape lines with superior tocopherol content and composition.

## Introduction

Vitamin E is an essential micronutrient for humans and mammals, which have no ability to synthesize it. Vitamin E only accumulates in photosynthetic organisms, in which it consists of tocopherols and tocotrienols, a group of amphipathic molecules composed of a polar chromanol head group derived from the shikimate (SK) pathway and a polyprenyl lipophilic side chain from the methylerythritol phosphate (MEP) pathway and chlorophyll degradation; these amphipathic molecules differ in the degree of saturation of their aliphatic tails. Within the tocopherols and tocotrienols, four forms (α, β, γ, δ) vary in the number and position of methyl groups on the chromanol ring. The α-tocopherol form is considered to have the highest nutritional value for humans and livestock [1]–[4]. Tocopherols are the major form of vitamin E in the seeds of dicots, but tocotrienols exist widely in the seeds of monocots [5], [6]. Besides its nutritional value, vitamin E is also a major natural antioxidant in seed oils, making it critical for polyunsaturated fatty acid stability [5]. Due to its benefits for health and oil quality, improving the content or composition of vitamin E in staple crops has been a major aim of crop breeding [7].

The vitamin E biosynthetic pathway has been well elucidated in the model species *Arabidopsis thaliana* and *Synechocystis* sp. PCC6803 [1], [6], [8]–[14]. Most of the genes encoding the key enzymes of the core biosynthetic pathway have been identified and functionally characterized, and select genes have been transformed and overexpressed individually or collectively in various plants. For example, the γ-tocopherol methyltransferase gene *VTE4* has been overexpressed in the seed of *A*. *thaliana* to elevate α-tocopherol content (αTC), and co-overexpression of the 4-hydroxyphenylpyruvate dioxygenase gene *PDS1* and the homogentisate phytyltransferase gene *VTE2* has been carried out in rapeseed (Brassica napus L.) seeds to increase the total tocopherol content (TTC) [15], [16]. However, the results were inconsistent between experiments. It is possible to alter the tocopherol composition (TCO) by seed-specific overexpression of *VTE4*, resulting in nearly complete conversion of γ-tocopherol to α-tocopherol in the seeds of *A. thaliana*. In contrast, it is relatively difficult to significantly increase the TTC in rapeseed. The most successful attempt at increasing TTC occurred in rapeseed, with nearly two-fold enhancement of TTC after co-transformation with *A*. *thaliana* genes *HPPD* and *VTE2* [11], [15]–[18]. These observations indicate that the tocopherol biosynthetic pathway and its regulation are more complex than supposed to date.

Edible oils are major dietary sources of vitamin E [19]. Rapeseed is one of the most important oil crops, and is grown mostly in temperate climates worldwide. The most abundant types of vitamin E in rapeseed oil are α- and γ-tocopherol, as well as a small proportion of δ-tocopherol [20]–[22]. Seeds of rapeseed vary widely in terms of tocopherol content and composition. In a study by Goffman and Becker (2002) in a germplasm collection of 87 rapeseed accessions, the TTC in seeds of rapeseed ranged from 182 to 367 ppm [19]. Fritsche *et al.* (2012) reported an even broader range of variation (197.5 to 460.1 ppm) for TTC in one of the investigated germplasm collections [23]. These variations provide an incentive for breeding high-tocopherol varieties having superior tocopherol content. The genetic mechanism for this large variation remains unclear.

Quantitative genetic approaches, which map quantitative trait loci (QTL) onto linkage maps or which detect associations between markers and phenotypes, are powerful methods to dissect complex metabolic traits [24]. As an example, Wentzell *et al*. (2007) found that all glucosinolate expression QTL coincided with glucosinolate metabolic QTL in *A. thaliana*, indicating that metabolic QTL regions may encompass candidate genes for the respective metabolic pathway [25]. Gilliland *et al.* (2006) detected *Arabidposis* QTL associated with tocopherol content and composition, and identified 14 QTL affecting tocopherol content and composition in seeds. Of these 14 QTL, five contained tocopherol biosynthesis candidate genes, implying that QTL mapping has the power to uncover genetic variations that previously had not been well characterized, with the exception of variations caused by mutations of known genes [26]. To date, a single study of QTL mapping associated with seed tocopherol content and composition has been carried out in rapeseed. Marwede *et al.* (2005) used a segregating doubled haploid population of rapeseed to identify eight QTL distributed on six linkage groups. Furthermore, Marwede *et al.* demonstrated that seed tocopherol content and composition were significantly affected by genotype, environment, and strong genotype×environment interactions. The authors reasoned that only a small number of genes are involved in tocopherol biosynthesis. However, the use of only one doubled haploid population with quite low phenotypic variation was a limitation of that investigation [27].

Association analysis based on linkage disequilibrium (LD) is another strategy for illustrating quantitative inheritance. There are two association analysis approaches: candidate-gene association analysis and genome-wide association study (GWAS). Candidate-gene association analysis, which is applicable to relatively simple or well-dissected biosynthesis pathways, is based on the notion that sequence variations within candidate genes cause phenotypic variation. In contrast, GWASs rely on a very high marker density to tag any region of the genome [28]. This method has been applied in many crops due to the benefit of detecting phenotype-associated polymorphisms without constructing mapping populations [29], [30]. One limitation of association analysis is that collections of genotypes, especially large collections with abundant genetic diversity, may have different population structures caused mainly by local adaptation or a variety of selections and familial relatedness from recent co-ancestry, which can give rise to spurious associations [31]. Nevertheless, a series of statistical programs and methods, such as STRUCTURE, SPAGeDi, unified mixed-model, and principal component analysis (PCA), have been developed to overcome this limitation [32]–[34]. Rare sequence variants are another problem. The basic theory for GWAS is that common genetic variations explain quantitative trait variations; rare alleles will therefore decrease the detection power of GWAS [35]. The extent of LD determines the resolution of an association analysis. A high level of LD decay implies low resolution, but a low level of LD decay means that a higher marker density is needed [36], [37]. Two GWASs previously investigated population structures and LD to demonstrate that rapeseed populations are highly structured and that LD decays rapidly [38], [39].

Rapeseed and *A. thaliana* share a common ancestor that existed 14–20 million years ago [40]. Comparative alignment analysis between *A. thaliana* and rapeseed and *in silico* mapping of *A. thaliana* genes onto the rapeseed linkage map previously enabled efficient QTL mapping in rapeseed [41]. In contrast to *A. thaliana*, rapeseed is a polyploid species with a genome that is ten times larger [42]. On average, 2–6 copies of each *A. thaliana* gene occur in the rapeseed genome [43], [44]. Correspondingly, the number of tocopherol biosynthesis genes in rapeseed is expected to be much higher than that in *A. thaliana*.

In this report, we describe the genetic architecture of seed tocopherol content and composition in rapeseed using bi-parental populations and a worldwide panel of rapeseed accessions. This combination of QTL and association mapping analysis provides a detailed picture of the network of tocopherol biosynthesis genes in rapeseed.

## Results

### Phenotypic Diversity and Trait Correlation

In a first experiment, a Tapidor×Ningyou7 doubled haploid population (TNDH), its reconstructed F_2_ (RC-F_2_) population, and both parents were grown under four environments over two growing seasons. Moderate differences were observed between Tapidor and Ningyou7 for αTC, γ-tocopherol content (γTC), TTC, and TCO. The winter-type parent Tapidor had a consistently higher TTC than the semi-winter-type parent Ningyou7; such that the TTC ranged from 349 ppm to 355 ppm in Tapidor but varied from 330 ppm to 331 ppm in Ningyou7 through different environments (Figure S1). Broad variations occurred in tocopherol content and composition, with normal or near-normal distributions, and extreme values at both ends of the distributions exceeded the extreme values of both parental distributions, indicating transgressive segregation (Figure S2A–P). Analysis of variance (ANOVA) revealed highly significant (*P*<0.0001) genetic and environmental effects for tocopherol content and composition. Heritabilities were considerably high, ranging between 0.65 and 0.78 for tocopherol traits (Table 1). Furthermore, we found high genetic correlations between γTC and TTC (0.91) as well as TCO (0.78; Table 2).

**Table 1:** Statistical parameters and variance components of trait performance in the TNDH population, RC–F_2_ population and the associationpanel of 142 accessions.

Parameters	α-toc	γ-toc	t-toc	α-toc/γ-toc
*TNDH population*				
Mean (ppm)	122	220	342	0.55
Min (ppm)	59	107	166	0.48
Max (ppm)	170	356	526	0.64
*σ^2^_g_*	126.39***	395.38***	736.87***	0.0026***
*σ^2^_g_*_x*e*_	104.54***	649.73***	1098.9***	0.0022***
*σ^2^_e_*	6.99***	7.25***	13.56***	0.00019***
*h^2^*	0.78	0.65	0.67	0.77
*RC-F_2_ population*				
Mean (ppm)	137	232	369	0.59
Min (ppm)	109	167	293	0.44
Max (ppm)	183	315	493	0.75
*Association panel*				
Mean (ppm)	160	177	340	0.96
Min (ppm)	84	85	182	0.33
Max (ppm)	286	281	460	2.14
*σ^2^_g_*	100.35***	103.97***	92.63***	0.091***
*σ^2^_g_*_x*e*_	21.12***	41.09***	91.39***	0.037***
*σ^2^_e_*	8.8***	10.58***	17.22***	0.0049***
*h* ^2^	0.81	0.85	0.65	0.82

Abbreviations: α-toc, α-tocopherol content; γ-toc, γ-tocopherol content; t-toc, total tocopherol content; α/γ, tocopherol composition.

*** *P*<0.0001.

**Table 2:** Genetic correlation coefficients of trait performance in the TNDH population and association panel.

Population	trait	α-toc	γ-toc	t-toc	α/γ	oil content
*TNDH population*	α-toc	/	0.51**	0.78**	0.53**	/
	γ-toc	/	/	0.91**	−0.44**	/
	t-toc	/	/	/	−0.02	/
*Association panel*	α-toc	/	−0.71**	0.33**	0.93**	−0.01
	γ-toc	/	/	0.43**	−0.91**	0.39**
	t-toc	/	/	/	−0.02	0.49**

** *P*<0.001.

In a second experiment, 142 rapeseed accessions from all over the world were grown under two environments over two growing seasons. Large variations in tocopherol content and composition were observed. Most remarkably, the TTC varied from 181 ppm to 460 ppm (Figure S2Q–X). As in the TNDH population, genetic variances were highly significant. The broad-sense heritabilities were even higher, ranging between 0.65 and 0.85 (Table 1). Interestingly, genetic correlations were considerably different from each other, as in the TNDH population. The highest correlation was detected between αTC and TCO (0.93), while the correlation between γTC and TTC was only 0.43 (Table 2).

### QTL Mapping for Tocopherol Content and Composition in the TNDH and RC-F_2_ Populations

We calculated QTL for αTC, γTC, TTC, and TCO. Phenotypic data were taken from the TNDH population (three environments) and from the RC-F_2_ population (one environment). A total of 57 QTL were detected, with 53 QTL in the TNDH population and four QTL in the RC-F_2_ population. These 57 QTL explained 5.0–20.3% of the phenotypic variation in tocopherol content and composition, and 70% of the QTL exerted modest effects, with *R^2^*<10%. For most of the QTL (63%), Tapidor alleles caused an elevation in tocopherol contents which was in accordance with the higher tocopherol contents of this parent (Table S1). All QTL were distributed across ten linkage groups (A2, A3, A5, A7, A9, A10, C2, C3, C8, and C9), with four main QTL clusters on A3, A7, A9, and C3 (Figure S3). QTL clusters on linkage groups A7 and A9 (*qTOC.A7* and *qTOC.A9*), which were mainly associated with αTC and TTC, were detected in both populations.

Doubled haploid plants carrying the positive Tapidor QTL alleles (*qTOC.A7* and *qTOC.A9*) were selected for backcrossing with the recurrent parent Ningyou7, which carries the negative alleles in these QTL regions. We investigated 63 BC_4_F_2_ lines and 265 BC_4_F_3_ lines via molecular markers located in or near *qTOC.A7* and *qTOC.A9*. We confirmed that these lines carried the positive QTL alleles from Tapidor in *qTOC.A7* and *qTOC.A9*. The lines were grown in the field and the greenhouse, and tocopherol contents were measured. As a result, 14 nearly isogenic lines (NILs) containing the Tapidor QTL alleles had significantly higher αTC or TTC (Table 3 and Figure S4).

**Table 3:** Trait performance of nearly isogenic lines (NILs) homozygous for QTL on A7 and A9.

NIL‡	QTL	α-toc§ (ppm)	γ-toc§ (ppm)	t-toc§ (ppm)
A07				
6C309-7	*uqTOC.A7-1*	128±4	264±6	392±5
6C309-17	*uqTOC.A7-1*	137±4	274±7	410±11
6C309-9	*uqTOC.A7-1*	133±5	274±7	407±12
6C309-21	*uqTOC.A7-2*	138±3	259±8	397±12
6C309-16	*uqTOC.A7-3*	132±3	271±8	403±11
6C309-20†	*qTOC.A7*	155±7	293±9	448±16
6C309-3†	*qTOC.A7*	179±6	288±7	467±13
Ningyou7		116±3	240±6	356±7
A09				
9C57-7	*uqTOC.A9-1*	228±6	158±9	386±12
9C45-10	*uqTOC.A9-2*	201±9	196±7	397±9
9C51-2*	*qTOC.A9-3,4,5*	217±9	199±5	415±12
9C36-9†	*qTOC.A9*	272±8	185±7	457±8
9C37-5†	*qTOC.A9*	223±9	185±9	407±10
Ningyou7		192±3	152±4	343±2

Both recurrent parents of the BC_4_F_2_ population for QTL on A7 and the BC_4_F_3_ population for QTL on A9 are listed as references. Means and standard deviation are shown from 10 plants.

‡ NILs and the recurrent parent Ningyou7.

† The NIL contains the whole QTL cluster.

* The NIL contains part of the QTL cluster.

§ All NILs were significantly different (*P*<0.01, ANOVA) compared with recurrent parent Ningyou7.

The meta-analysis, which was used to integrate QTL for various traits in different environments into unique QTL, was carried out in two steps. First, QTL detected for the same trait in different environments were integrated into nonredundant QTL. In the second step, QTL for different traits were merged into unique QTL. We identified 47 nonredundant QTL, of which 15, 11, 12, and nine were associated with αTC, γTC, TTC, and TCO, respectively (Table S2). Further analysis of the nonredundant QTL revealed 33 unique QTL in the TNDH and RC-F_2_ populations. Of these, 16 unique QTL were found to exert pleiotropic effects, as each of them was associated with two or more tocopherol traits (Table 4).

Investigation of epistatic interactions revealed six significantly interacting pairs of loci controlling tocopherol content and composition in the TNDH and RC-F_2_ populations in various environments. Six QTL were involved in these interactions, including one QTL/QTL interaction, four QTL/non-QTL interactions, and one non-QTL/non-QTL interaction. Interactions were mainly additive×additive. TTC and γTC were significantly affected by additive×additive epistatic effects, which explained 9.5–20.8% of the genotypic variance (Table S3).

### Genetic and *in silico* Mapping of Candidate Genes Associated with Tocopherol Biosynthesis

We selected five *B. napus* genes with high similarity to *A. thaliana* genes *VTE2*, *VTE3*, *VTE4*, *VTE5*, and *PDS1* as candidate genes for genetic linkage mapping. These genes were mapped to five linkage groups using the existing *B.* napus map (TNDH population). Interestingly, three of these genes co-localized with previously mapped tocopherol QTL (Figure S5). Comparative alignment of *B. napus* and *A. thaliana* was implemented based on 375 markers with sequence information that can be aligned with *A. thaliana* sequences in the *Arabidopsis* information resource (TAIR, http://www.arabidopsis.org/; Table S4). Subsequently, we searched the TAIR database for genes underlying SK, MEP, chlorophyll degradation, and tocopherol core biosynthesis pathways in *A. thaliana*. These *A. thaliana* genes were aligned to the TNDH linkage map based on the comparative alignment of *B. napus* and *A. thaliana* and 14 *A. thaliana* genes located in seven unique QTL. Twelve genes were responsible for homogentisate and phytyl diphosphate biosynthesis, two precursors of tocopherol biosynthesis (Table 4 and Table S4).

### Whole-genome Association Analysis

**Figure 1:**
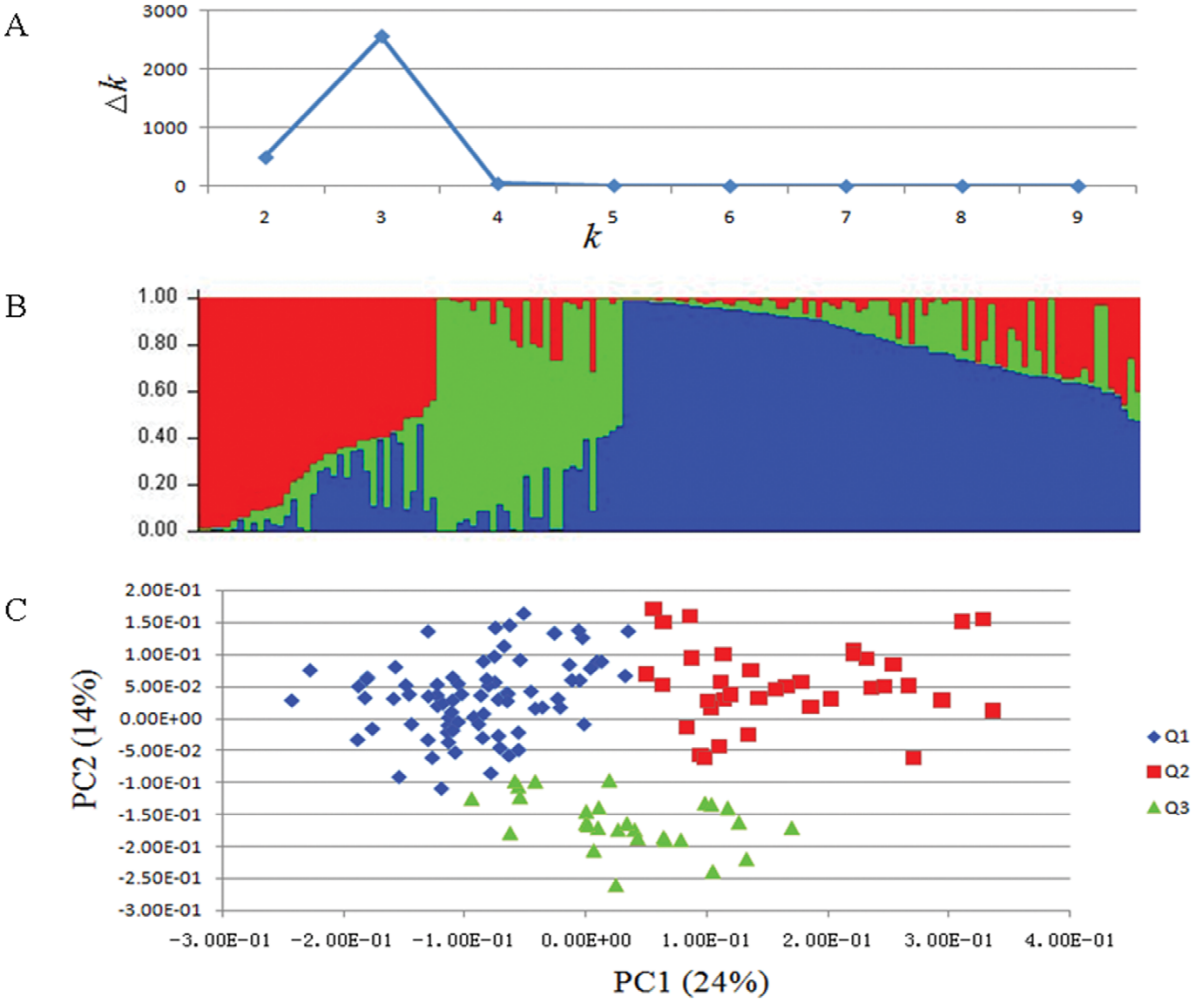
Population structure of the *B. napus* panel of 142 accessions from a worldwide collection. The program package STRUCTURE2.2 was used, and a principal component analysis was performed via NTYSpc. (A) Estimation of the number of subpopulations for *k* ranging from 1 to 10 by calculating Δ*k*. The calculation procedure was as presented by Evanno *et al*. (2005). (B) STRUCTURE result for *k* = 3. (C) Principal component analysis result. In (B) and (C): subpopulation 1 (Q1), blue; subpopulation 2 (Q2), red; subpopulation 3 (Q3), green.

Considerable phenotypic variation in tocopherol content and composition was observed in the rapeseed association-mapping panel of 142 accessions in two environments (Table 1 and Figure S2Q–X). Forty simple sequence repeat markers, evenly distributed across the TNDH linkage groups, were used to infer population structure to derive 102 polymorphic loci (Table S5). We determined the population structure by using the program STRUCTURE and by PCA [31], [33]. Program STRUCTURE revealed three subgroups with *k* = 3 identified as the best turning point with the highest Δ*k* in the association panel; most *B. napus* accessions were assigned to subgroup 1, 36 accessions were in subgroup 2, and 28 accessions were assigned to subgroup 3 (Figure 1A and Figure 1B ). Subgroup 1 consisted of nearly all winter-type accessions, including the parent of the TNDH population (Tapidor). In contrast to subgroup 1, most accessions in subgroup 3 were annuals (spring type). Subgroup 2 contained both winter- and spring-type accessions, as well as semi-winter-type accessions such as the other parent of the TNDH population (Ningyou7; Table S6). The population substructure identified by program STRUCTURE was confirmed by the analysis of PCA (Figure 1C).

**Table 4:** Unique QTL for tocopherol content and composition calculated with meta-analysis in the TNDH and RC-F_2_ populations in all environments.

QTL	Trait	Pos(cM)	CI (cM)	LOD	*R* ^2^(%)	A	Candidate At loci by*in silico* mapping	*B. napus* loci bygenetic mapping
*uqTOC.A2-1*	α	89.81	86–93	3.13	6.17	−8.41	At1g64970, At1G63970,At1G19670	*BnaA.VTE4.a* (At1g64970*)
*uqTOC.A2-2*	α	99.11	98.2–100.2	3.52	6.78	−4.34		
*uqTOC.A2-3*	t	110.51	107.5–114.3	5.29	10.21	−5.23		
*uqTOC.A3-1*	α, α/γ	34.61	33.8–35.42	2.95–6.0	5.28–10.74	0.02–3.9		
*uqTOC.A3-2*	α, α/γ	39.33	38.16–40.49	3.53–3.65	6.5–6.86	0.02–3.74		
*uqTOC.A3-3*	α, α/γ	43.21	42.55–43.87	3.23–3.87	6–8.55	0.02–6.16		
*uqTOC.A3-4*	α/γ	47.91	47–50.9	2.55	5.13	−0.03		
*uqTOC.A5-1*	γ, *t*	36.71	33.4–48	2.74	6.13	−11.36		
*uqTOC.A5-2*	*t*	54.91	52.8–57.02	3.32–3.66	5.94–6.54	1.68		
*uqTOC.A7-1*	α, *t*	19.6	18.48–20.72	3.33–9.04	6.71–20.33	3.66–10.85	At2g18950	*BnaA.VTE2.a* (At2g18950*)
*uqTOC.A7-2*	α, *t*	25.63	24.8–26.47	2.52–9.53	5.03–18.27	3.14–10.66		
*uqTOC.A7-3*	α, t	31.21	28.73–33.69	4.17–9.04	8.13–17.54	5.47–5.86		
*uqTOC.A9-1*	γ	53.21	51.9–54.3	3.78	8.58	9.38		
*uqTOC.A9-2*	γ, *t*	65.54	63.72–67.36	7.17–8.52	12.86–16.72	11.42–14.95		
*uqTOC.A9-3*	γ, *t*	71.04	69.69–74.38	3.53–5.1	8.9–11.31	9.62–12.23		
*uqTOC.A9-4*	γ, *t*	77.81	76.14–79.48	4.2–4.8	9.02–9.72	6.43–12.23		
*uqTOC.A9-5*	γ, t	80.34	79.03–81.65	4.2–5.55	9.02–11.35	6.43–12.23		
*uqTOC.A9-6*	t	86.84	84.34–89.23	5.02	10.35	10.32	At1g29410, At1G74470	
*uqTOC.A9-7*	γ	91.21	89.9–101.4	3.33	6.95	5.65	At1g22410	
*uqTOC.A10-1*	α/γ	34.61	30.5–37.2	4.16	8.95	0.03	At1g06590, At1g29410	*BnaA.PDS1.c* (At1g06590*)
*uqTOC.A10-2*	γ, α/γ	43.94	42.05–45.84	3.34–4.63	8.31–9.01	−11.96		
*uqTOC.C2-1*	α	49.51	44.1–53.4	3.37	6.67	−3.65		
*uqTOC.C2-2*	α	56.01	53.4–61.6	3.28	6.92	−3.68		
*uqTOC.C3-1*	α, α/γ, γ	25.27	18.94–31.61	2.91–7.38	6.21–16.92	−17	At4g33510, At4g34350,At4g36810, At4g38460	
*uqTOC.C3-2*	α, γ, *t*	42.01	39.71–44.31	4.05–7.38	8.41–16.92	3.25	At5g53970	
*uqTOC.C3-3*	α	46.71	46.1–50.5	6.05	12.56	−4.98		
*uqTOC.C3-4*	α, t	52.51	49.82–55.2	3.36–5.05	6.85–10.82	3.49		
*uqTOC.C8-1*	γ, t	36.16	29.74–43.64	2.58–3.82	5.08–7.34	5.17		
*uqTOC.C8-2*	γ	54.31	48–59.1	3.25	6.81	−5.53		
*uqTOC.C9-1*	α	52.01	51.7–53.1	3.2	6.97	4.33		
*uqTOC.C9-2*	α/γ	85.91	82.1–87.7	3.91	8.14	0.02		
*uqTOC.C9-3*	α/γ	92.01	90.9–99.9	6.68	13.01	0.02		
*uqTOC.C9-4*	α	102.51	100–105.5	4.79	9.25	4.22		

At, *A. thaliana*; α, α-tocopherol content; γ, γ-tocopherol content; t, total tocopherol content; α/γ, tocopherol composition; Pos, peak position; CI, confidence interval of the QTL region; LOD, LOD score; *R* ^2^, explanation of phenotypic variation; A, additive effect; WH, Wuhan 2003–004 growing season; JZ, Jingzhou 2003–2004 growing season; WN, Weinan 2004–2005 growing season.

* The *A. thaliana* gene is homologous to the corresponding *B. napus* gene.

**Figure 2:**
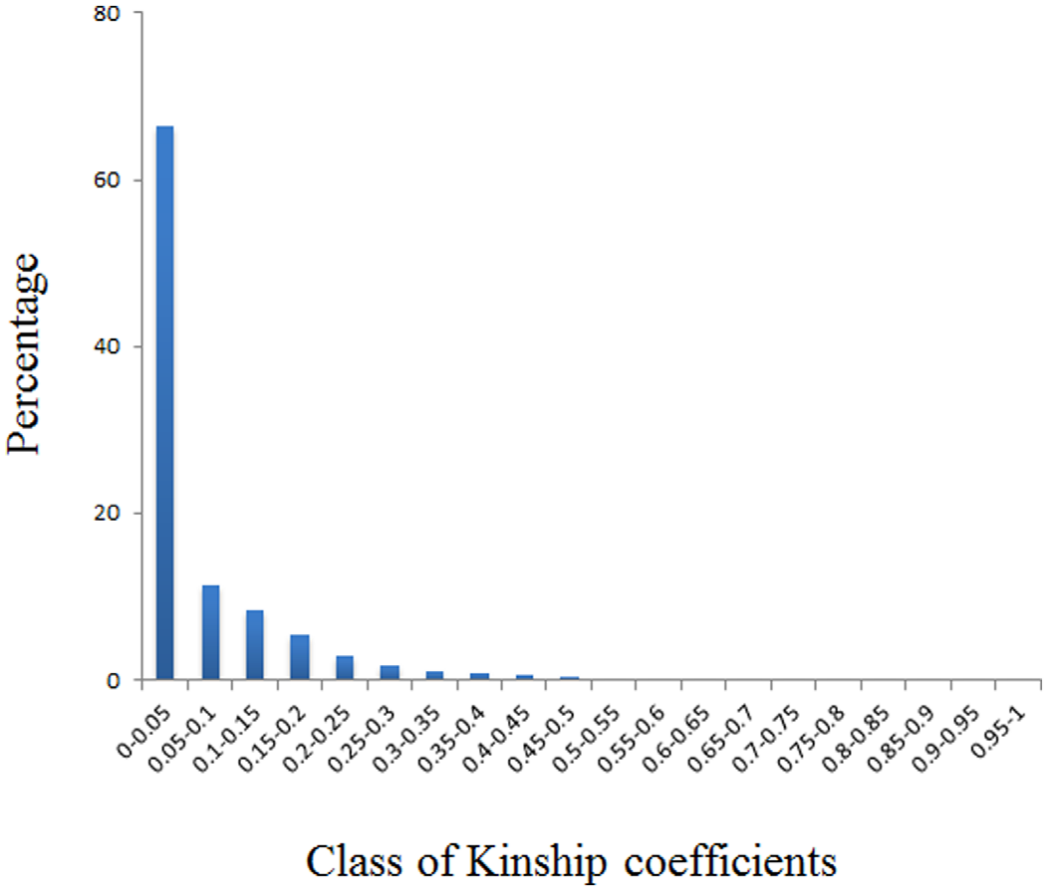
Distribution of kinship coefficients between 142 *B. napus* accessions. Kinship coefficients values from SPAGeDi estimates were calculated with 101 markers.

Next, we calculated the familial relatedness (kinship) between accessions by utilizing the same set of markers as the subsequent association analysis. Relatedness was calculated with 101 markers with 224 polymorphic loci. More than 65% of the pairwise kinship values ranged between 0–0.05, indicating a low level of relatedness between varieties (Figure 2). When we tested the effects of population structure (Q) and kinship (K) on phenotypic variation, we found that both parameters exerted a significant effect on phenotypic variation, e.g. the Q effect and the K effect explained 39.7% and 47.1% of the αTC variation, respectively (2009–2010 growing season in Jingzhou; Table 5).

**Table 5:** Effects of population structure and kinship on phenotypic variation.

Trait	Q effect (%)	K effect (%)
08–09		
total-toc	6.43	18.70
α/γ	36.30	46.10
α-toc	24.60	33
γ-toc	30.70	32.40
09–10		
total-toc	4.18	14.50
toc-composition	41.40	31.40
α-toc	39.70	47.10
γ-toc	36.40	24

Q, population structure; K, kinship; 08–09, 2008–2009 growing season in Jingzhou; 09–10, 2009–2010 growing season in Jingzhou.

**Figure 3:**
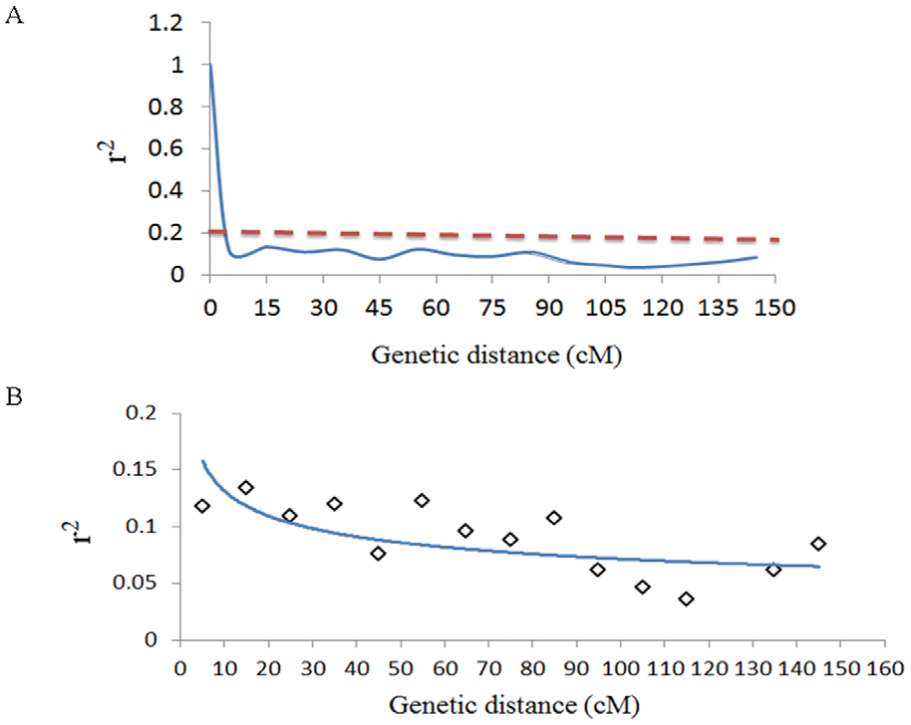
Plot of LD extent (*r^2^*) against the genetic map distance (cM) over the whole genome. (A) An overview of the LD decay over the whole genome. (B) An enhanced view of the the LD decay overthe whole genome. Blue solid line, nonlinear regression trend line of *r^2^* against the genetic map distance. Red dashed line, threshold as the 95% quantile of the *r* ^2^ value among unlinked loci pairs.

The extent of LD decay in the association panel was evaluated using pairwise combinations of 81 markers derived from the TNDH linkage map (Table S5). LD decay decreased within 2 cM over the whole genome and within 1 cM on chromosome A9 (Figure 3 and Figure S6).

**Figure 4:**
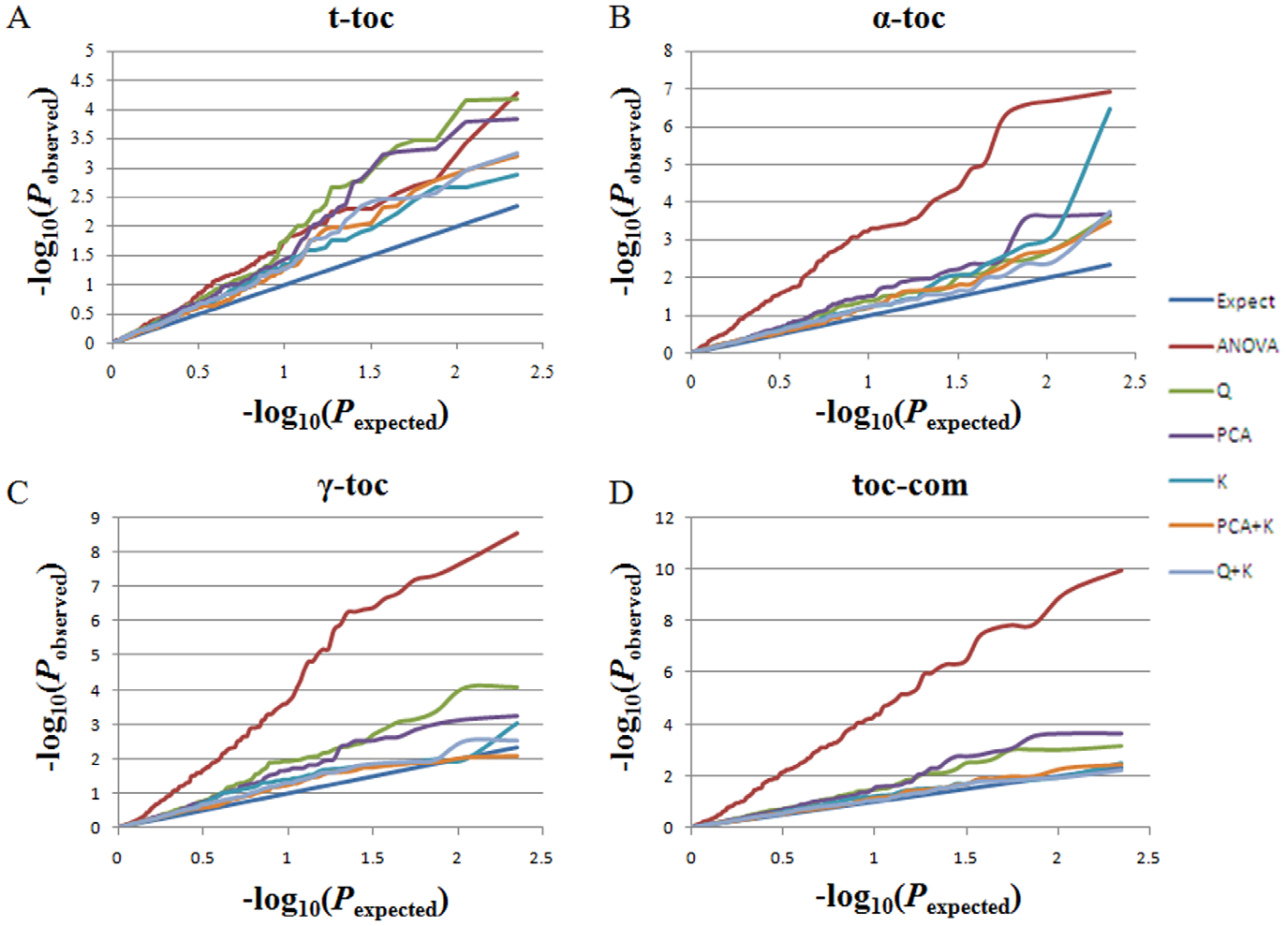
Quantile-quantile plots for tocopherol-associated traits constructed via six methods. (A) Total tocopherol content. (B) α-tocopherol content. (C) γ-tocopherol content. (D) Tocopherol composition. Horizontal axis, −log_10_−transformed expected *P* values. Vertical axis, −log_10_−transformed observed *P* values. Expect, expected *P* values under the null distribution; ANOVA, observed *P* values by analysis of variance; Q, observed *P* values by generalized linear model with Q matrix; PCA, observed *P* values by general linearized model with the principal component matrix; K, observed *P* values by multilevel modeling with the K matrix; PCA+K, observed *P* values by multilevel modeling with the K matrix and the principal component analysis matrix; Q+K, observed *P* values by multilevel modeling with the K matrix and the Q matrix.

**Figure 5:**
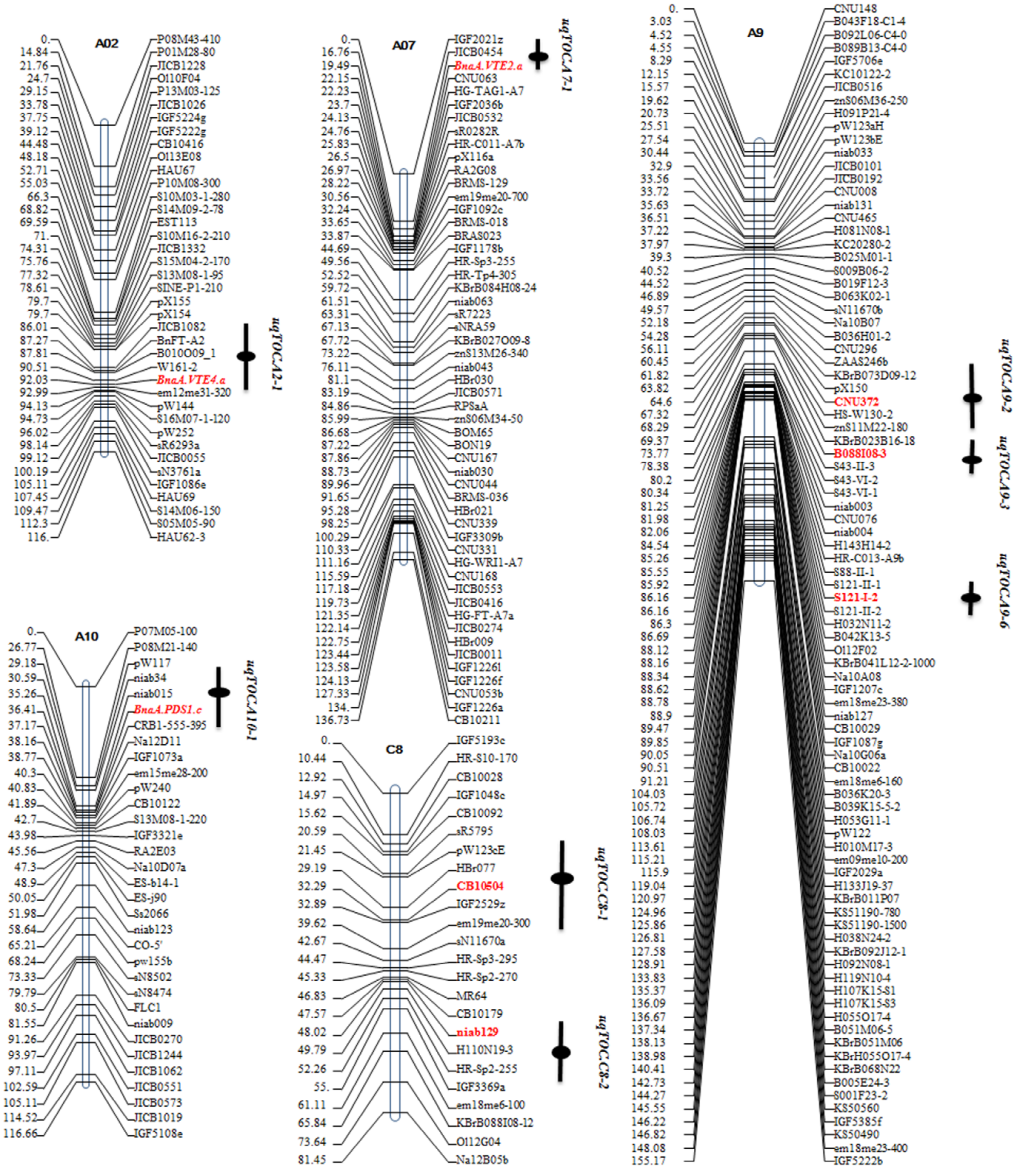
Comparison of QTL mapping and association analysis results. Red, markers significantly associated with tocopherol-related traits detected in both growing years. Black lines, quantitative trait loci confidence interval. Black solid circles, peak position of the quantitative trait loci.

For GWAS, a total of 101 markers were analyzed for association with four tocopherol-related traits (αTC, γTC, TTC, and TCO) via six models (ANOVA, Q, PCA, K, PCA+K, and Q+K). Earlier, we had determined which model best fit each trait. The K model was the best fit for TTC, while the Q+K model best fit αTC. K, PCA+K, and Q+K explained γTC and TCO equally well (Figure 4). Therefore, the K model was selected for TTC while the Q+K model was chosen for αTC, γTC, and TCO. Sixty-one loci were significantly associated with four tocopherol-related traits in data from two growing years. Interestingly, 11 of these loci were located within the confidence intervals of the respective QTL regions, four within *qTOC.A9*, two within *qTOC.C3* and *qTOC.C8*, and one within *qTOC.A2*, *qTOC.A7*, and *qTOC.A10* (Table S7). Seventeen of 61 associated loci were significantly associated with tocopherol-related traits in data from two growing years, while eight of them were located in the QTL regions (*uqTOC.A2-1*, *uqTOC.A7-1*, *uqTOC.A9-2*, *uqTOC.A9-3*, *uqTOC.A9-6*, *uqTOC.A10-1*, *uqTOC.C8-1*, and *uqTOC.C8-2*). Most interestingly, three markers derived from tocopherol candidate genes (*BnaA.VTE4.a*, *BnaA.VTE2.a*, and *BnaA.PDS1.c*) were significantly associated with tocopherol content and composition in both growing years and also co-localized with QTL *uqTOC.A2-1*, *uqTOC.A7-1*, and *uqTOC.A10-1* (Table 6 and Figure 5).

**Table 6:** Associated markers identified in both two years’ genome wide association studies of tocopherol content and composition with 101 markers.

Marker	LG	Environment‡	α-toc#	γ-toc#	t-toc#	α-toc/γ-toc#
B088I08-3†	A09	08JZ		0.0168***	0.0676***	
		09JZ		0.0259***	0.0654***	
Ol12B03	C02	08JZ	0.018***		0.0566*	
		09JZ			0.038**	
sORA43	C02	08JZ	0.0225*	0.0201*	0.0479**	
		09JZ	0.0138*			
CNU372†	A09	08JZ		0.0127*	0.0448**	
		09JZ		0.0295*	0.0277*	
Na12C08	C01	08JZ		0.0189*	0.0244*	
		09JZ		0.0372***		0.0447***
CNU235	A01	08JZ	0.0199*		0.0232*	
		09JZ			0.0296*	
CNU296	A09	08JZ		0.0166*		
		09JZ		0.0206*		
CB10504	C08	08JZ		0.0161*		0.0079*
		09JZ		0.0259*		
*BnaA.VTE2.a* †	A07	08JZ	0.0193*	0.0161*		
		09JZ	0.0174*			
niab129†	C08	08JZ		0.0145*		
		09JZ			0.051**	
OL11H09	C02	08JZ		0.013*		0.0146*
		09JZ				0.0182*
niab047†	A09	08JZ	0.0583***			
		09JZ	0.0135*			
S121-I-2†	A09	08JZ	0.0347**			0.0088*
		09JZ		0.0506**		
*BnaA.VTE4.a* †	A02	08JZ	0.0186*			
		09JZ	0.0105*			
*BnaA.PDS1.c* †	A10	08JZ				0.0208**
		09JZ		0.0259*		0.027*
KBrB068N22	A09	08JZ				0.0136*
		09JZ		0.0293*		0.0209*
BES40		08JZ				0.0105*
		09JZ				0.0202*

LG, linkage group.

† Marker co-localized with detected quantitative trait loci.

‡ 08JZ indicates the 2008–2009 growing season in Jingzhou and 09JZ indicates the 2009–2010 growing season in Jingzhou.

# Explanation of phenotypic variation.

* *P*<0.05.

** *P*<0.01.

*** *P*<0.001.

## Discussion

### Tocopherol Variation in *B. napus* Seeds

Plant oil-derived tocopherols are an important source of vitamin E, which is a necessary micronutrient for human health. In this study, tocopherol content and composition were tested in a doubled haploid population, its derived RC-F_2_ population, and a panel of 142 *B. napus* accessions. A broad range of phenotypic variation was observed, which is largely in accordance with previous results [45], [46]. For all four traits measured, the phenotypic variation of the doubled haploid population largely exceeded both parents. Thus, the transgressive variation for tocopherol traits in *B. napus* has great potential for breeding rapeseed varieties with improved tocopherol characters.

Here we present a refined genetic analysis of tocopherol characters in *B. napus*. Previously, the heritabilities of tocopherol characters were reported to be low. Marwede *et al.* (2005) calculated rather low broad-sense heritabilities ranging from 0.23 for αTC to 0.5 for γTC, with 0.41 and 0.42 for TTC and TCO, respectively, resulting from significant genotype × environment interactions [20], [27], [46]. In our study, high broad-sense heritabilities were calculated for all four traits with the doubled haploid population as well as with the association panel, although strong genotype×environment interactions and environmental effects were detected. Thus, our observations imply that genetic variation was the main contributor to tocopherol variability, and that these traits are suitable for application in *B. napus* breeding.

As expected, significant genetic correlation coefficients were calculated between tocopherols and tocopherol-associated traits in the doubled haploid population and the association panel. The significant genetic correlation (0.91) between γTC and TTC in the TNDH population was consistent with previous reports, such as Marwede *et al*.’s (2004 and 2005) values of 0.91 and 0.92 in *B. napus* [20], [27]. However, a significant genetic correlation (0.51) was detected between αTC and γTC in the doubled haploid population that had not been reported before. The genetic correlation in the association panel was not in accord with the correlation in the TNDH population; for example, the correlation between αTC and γTC was positive in the TNDH population but negative in the association panel, indicating that the complex population structure of the association panel may affect the evaluation of genetic effects. The prominent genetic correlations between γTC and oil content as well as between TTC and oil content indicate that an increase in oil content can result in elevated TTC in *B. napus* seeds and vice versa.

### Genome-wide QTL Detection and Homologous Gene Mapping

Fifty-seven QTL distributed on ten linkage groups were detected in the populations under study. Furthermore, meta-analysis revealed 33 unique QTL, of which 16 were pleiotropic. Thus, we detected considerably more QTL than a previous study that reported eight QTL related to tocopherol content and composition on six linkage groups in a doubled haploid population [27]. However, a comparison of QTL regions between these two populations was not possible due to the lack of anchor markers. By comparing the QTL distributions of these two populations, we demonstrated that four linkage groups (A3, A7, C3, and C9) carried QTL in both populations, with two linkage groups (A7 and C3) containing QTL associated with the same traits (Table S8). This observation suggests that QTL mapping results in bi-parental populations are subject to the variation between the two parents. Four QTL clusters were detected; *qTOC.A*3 was associated with αTC and TCO, *qTOC.A7* with αTC and TTC, and *qTOC.A9* was especially associated with γTC and TTC. These results are consistent with our genetic correlation analysis, in which high genetic correlations were detected between αTC and TCO (0.53), αTC and TTC (0.78), and γTC and TTC (0.91).

Epistasis is another main genetic factor underlying complex traits [47]. In this study, γTC, TTC, and TCO each had two interaction pairs. Significant additive×additive epistasis effects had been detected for γTC and TTC in both doubled haploid population and its derived RC-F_2_ population. These results imply the importance of epistasis effects in the genetic basis of γTC and TTC.

Rapeseed oil with high TTC is supposed to have good oil stability due to the antioxidant function of tocopherols, and high αTC oils have good nutritional value [48]. It is noteworthy that two QTL clusters, *qTOC.A7* associated with αTC and TTC and *qTOC.A9* with γTC and TTC, are most applicable for advancing tocopherol content and composition in seed of *B. napus*. Several NILs introgressed segments of the higher parent (Tapidor); plants derived from these two QTL clusters showed significant elevation of αTC and TTC compared with the recurrent parent Ningyou7, indicating that genetic variations underlying these QTL influenced tocopherol content. Further dissection of the genetic basis of these QTL will benefit the breeding of *B. napus* varieties with high αTC or TTC.

Thanks to the rapid development of genomic research in plants, the tocopherol-related biosynthesis pathway has been well characterized in recent decades. Many genes encoding the key enzymes of these pathways have been cloned and used for the genetic engineering of biofortified staple crops [3], [7], [9], [11], [15], [18], [49]–[56]. A candidate/homologous gene approach based on characterized genes in metabolism biosynthesis pathways was a strategy used for dissecting complex traits, a strategy that can also assist in the identification of the genes responsible for QTL [57], [58]. Here, 14 *A. thaliana* genes associated with tocopherol biosynthesis were mapped onto the TNDH linkage map and co-localized with seven unique QTL by *in silico* mapping. Subsequently, five *B. napus* genes homologous to *A. thaliana* in the core pathway of tocopherol biosynthesis were mapped onto the TNDH linkage map by genetic mapping, with three co-localized in QTL regions. Homologous genes located in QTL regions may provide information regarding the genetic variations underlying the QTL. On the other hand, we did not identify known genes in many of the detected QTL such as *qTOC.A9*, a major QTL cluster related to γTC and TTC. It has been suggested that novel genetic loci affect tocopherol contents in *B. napus* seeds, a hypothesis in accord with the observation that just five of 14 QTL contained known genes related to tocopherol biosynthesis in an investigation of tocopherol content and composition in two *Arabidopsis* recombinant inbred lines [26]. Furthermore, an alternative reason may be the polyploid nature of *B. napus* due to the fact that the *B. napus* genome is ten times larger than the genome of *A. thaliana*; on average, 2–6 copies of each *A.* *thaliana* gene can be found in *B. napus* [42]–[44].

### Genome-wide Association Analysis and Comparison with QTL Mapping

Association mapping based on LD analysis is a widely applied method for dissecting complex traits such as quantitative traits [59]. Although this method enjoys many advantages, such as the lack of a need to construct mapping populations and a large range of variations, several limiting factors exist [29].

Population structure is one of these limiting factors. Many statistical programs have been developed to resolve this problem, including the transmission disequilibrium test and the quantitative transmission disequilibrium test, which are used for family-based samples. Genomic control and structure association are applied to germplasm-based samples. Genomic control employs a large number of random markers to evaluate the effect of population structure, and assumes that this effect is fixed for all markers in the association analysis. However, genomic control may result in the loss of power for markers with unusual allele frequencies across ancestral populations [33], [60]–[63]. PCA, which describes the variation detected by all markers in terms of a few main component variables, has become a popular tool in population genetics [33], [64].

In this investigation, population structure was examined with PCA and the structure association-based program STRUCTURE. Both analyses assigned the association panel into three subgroups. Furthermore, we demonstrated that population structure contributed to phenotypic variation, with the exception of TTC. Although we detected a low level of pairwise relatedness, kinship significantly contributed to the phenotypic variation for all traits. This observation was consistent with previous studies. Atwell *et al*. (2010) carried out a GWAS of 107 phenotypes in *A. thaliana*, which indicated a marked reduction in the number of associations across phenotypes after correction for population structure in a parametric mixed model. Similarly, Wang *et al*. (2011) reported that population structure explained 53.6% of the flowering time variation in a panel of *B. napus* inbred lines [65], [66].

The extent of LD decay is an important factor in association analysis. In this study, the LD decayed rapidly over the whole genome as well as in individual linkage group A9. Our results support previous reports that the LD extended only for ∼2 cM in canola quality winter rapeseed and for 1 cM in a species-wide germplasm set of *B. napus*. Thus, high-resolution mapping can be obtained through association mapping in *B. napus* with a high density of markers [38], [39].

Dozens of statistical models have been developed for association analysis [29], [67]–[70]. Six widely used models were tested in this investigation, with the result that different traits fitted to different models. In this study, models that included ‘K’ performed better than models that only contained ‘Q’. This observation was consistent with the report that the *P*_observed_ value from the GLM model greatly deviated from the *P*_expected_ value, followed by the ‘Q’ model, while the *P*_observed_ value from the ‘K’ model and the ‘K+Q’ model were close to the *P*_expected_ value for TTC, plant height, and kernel length in a maize association panel [71]. After comparing various models, Stich *et al.* suggested that the ‘K+Q’ model was not only appropriate for association mapping in humans, maize, and *Arabidopsis*, but also for rapeseed, potato, and sugar beet, indicating that the ‘K+Q’ model can be applied widely to various species [72].

After correcting for population structure effects, 61 loci were significantly associated with tocopherol content and composition, and 17 loci were detected in the field experiments from both growing years. This observation implies that the association panel had abundant genetic variation for tocopherol content and composition, and that some genetic loci were stable across different environments, which should be useful in marker-assisted selection. Interestingly, 11 of the associated loci were co-localized with QTL regions, demonstrating the complementarity of association analysis and QTL mapping. The combination of these two approaches allowed us to exploit the abundant recombination events and mutations in the association samples during a long history as well as the statistical power of QTL mapping to detect the loci of rare alleles. Therefore, the joint use of linkage mapping and association mapping is a good alternative strategy for detecting genetic variations [28], [68]–[70], [72], [73].

### Conclusions

Our results demonstrate that the wide variations in tocopherol content and composition, the high levels of broad-sense heritabilities, and the complex but significant genetic correlations among tocopherol characters occurred not only in the bi-parental populations but also in the association panel. These observations suggest that there is tremendous genetic potential for improving the tocopherol content of *B. napus*. In addition, dozens of unique QTL and associated loci were detected in the bi-parental populations and the association panel for tocopherol content and composition from multiple environments, indicating that tocopherol content variation was caused by variations in many genetic loci. We used recombinant backcross lines to dissect the QTL regions of *qTOC.A7* and *qTOC.A9*. We discovered that lines with the introgressed segments of the Tapidor parent exhibited elevated αTC or TTC, showing that genetic variations underlying the QTL confidence intervals explained the variations in tocopherol content. Further analysis of these QTL will enable us to fully uncover the genetic basis for the variation in tocopherol content in *B. napus* seeds. Furthermore, approximately one-quarter of the unique QTL confidence intervals from *in silico* and genetic mapping identified homologous genes associated with tocopherol biosynthesis from *A. thaliana*, which provides information for QTL dissections. Finally, 17 significantly associated loci were identified in the data from both growing years; 11 of these loci were located in the QTL confidence intervals, which will be useful for breeding superior rapeseed varieties with high tocopherol content by marker-assisted selection. Taken together, QTL mapping, association analysis, and homologous gene mapping and alignment revealed a complex genetic network for tocopherol biosynthesis.

## Materials and Methods

### Plant Materials and Field Experiments

A segregating F_1_-derived doubled haploid population of 202 lines had been previously developed from a mating between a European winter cultivar, Tapidor, and a Chinese semi-winter cultivar, Ningyou7 [74]. Crosses had previously been made among doubled haploid lines to obtain a RC-F_2_ population with 436 lines [75], [76]. The TNDH population and its parents were planted in three natural environments at three locations in China (Wuhan, 114°19′E, 30°5′N./200 m; Weinan, 109°3′E, 34°5′N./800 m; Jingzhou, 112°11′E, 29°3′N./40 m) over two growing seasons (2003–2004, 2004–2005); the RC-F_2_ population was planted in one environment (Jingzhou) during 2003–2004. A panel of 142 *B. napus* accessions was planted in Jingzhou over two growing seasons (2008–2009, 2009–2010). For each experiment, the randomized complete block design was applied with three replications, and each plot included 30 plants of one genotype.

TNDH lines singled out by marker-assisted selection were used to develop NILs for *qTOC.A7* and *qTOC.A9*. A BC_4_F_2_ population was constructed for *qTOC.A7* in 2006 in the field of Wuhan, while a BC_4_F_3_ population was developed for *qTOC.A9* in 2009 in the greenhouse of Kiel. Ningyou7 was the recurrent parent for these two populations.

The plants grown in the field and greenhouse belonged to Huazhong Agricultural University and were grown only for DNA and RNA extraction and phenotypic evaluation. These field studies did not involve endangered or protected species.

### Tocopherol Content Measurement

A homogenous mixture of 30–50 mg of mature *B. napus* seeds were ground in a swing-mill (Geno/Grinder, Germany) with two 5-mm metal beads in the presence of 1000 µl *n*-heptane. Samples were incubated for 24 h in the dark at −20°C, and then centrifuged at 4°C for 15 min at 16,000×*g*; 50 µl of the clear supernatant were collected for high-performance liquid chromatography as previously described [18], [77]. Tocopherols were identified by comparison of retention time, and concentrations were calculated by comparison of the area values with values from exterior standard tocopherols (Merck, Germany). TTC was designated as the sum of αTC and γTC in air-dried seeds. TCO was the ratio of αTC to γTC.

### Statistical Analysis of Phenotypic Variance

Statistical analysis was conducted with SAS 8.0 [78]. Genotype, environment, and genotype×environment interaction variances in the TNDH population, the RC-F_2_ population, and the association panel were analyzed by ANOVA in the GLM. The broad-sense heritability was calculated with the formula *h* ^2^ = *σ* ^2^ *_g_*/(*σ* ^2^ *_g_*+*σ* ^2^ *_ge_*/n+*σ* ^2^ *_e_*/nr), where *σ* ^2^ *_g_*, *σ* ^2^ *_ge_*, *σ* ^2^ *_e_*, n, and r represent the genetic variance, the interaction variance of genotype×environment, the error variance, the number of environments, and the number of replications, respectively. Genetic correlation was calculated with the formula *r_G_* = *cov_Gxy_*/(*σ* ^2^ *_Gx_*×*σ* ^2^ *_Gy_*)^1/2^, where *cov_Gxy_*, *σ* ^2^ *_Gx_*, and *σ* ^2^ *_Gy_* were the genetic covariance and variance of the pair-wise traits, respectively. The significance of each genetic correlation was determined using a *t*-test of the correlation coefficients [76]. The mean value of each trait for all populations was used in subsequent QTL and association analyses.

### Linkage Map Construction and QTL Detection

A linkage map was developed with 344 molecular markers derived from the TNDH population [74]. Many molecular markers, including simple sequence repeats, restriction fragment length polymorphisms, sequence-related amplified polymorphisms, and sequence-tagged sites had previously been added to this core linkage map [75], [76]. In this report, a new linkage map spanning 2190 cM with 790 molecular makers was constructed by JoinMap3.0 (http://www.kyazma.nl/index.php/mc.JoinMap) and utilized in subseequent QTL analysis (Table S4). The program Windows QTL Cartographer 2.5 was used with the composite interval method for QTL mapping [79]. To define the QTL thresholds, the permutation test was carried out by randomly shuffling the trait values 1000 times under the condition of *P* = 0.05 [80]. LOD values of 2.47–3.26 for TNDH and 3.96–4.47 for RC-F_2_ were adopted to identify significant QTL. QTL detected in different environments were integrated into unique QTL in two steps with BioMercator 2.1 when their confidence intervals overlapped [81], [82]. QTL for the same trait in different environments were integrated into non-redundant QTL, then non-redundant QTL for different traits were integrated into unique QTL. The genetic effects of tocopherol, including single-locus and two-locus effects, in different environments were detected with QTLmapper 2.0 [83].

The QTL nomenclature in this report generally follows the description of Long *et. al* [75], [76]. The identified QTL were designated with the initial letter “*q,*” followed by an abbreviation for tocopherol (*TOC*), the linkage group name, and an abbreviation representing the various forms of tocopherol. The non-redundant QTL were named with the initial designation “*nq,*” followed by *TOC* and the linkage group. If more than one non-redundant QTL were detected in the same linkage group, the QTL name included an alphabetical letter. The unique QTL were designated with the initial letters “*uq,*” followed by *TOC*, the linkage group name, and the serial number of the QTL in the linkage group. QTL in the same linkage group were considered to be a QTL cluster, which was designated with the initial letter “*q,*” followed by *TOC* and the linkage group name.

### Comparative Alignment and *in silico* Mapping between *B. napus* and *A. thaliana*

Comparative alignment between *B. napus* and *A. thaliana* was based on the 375 molecular markers with sequence information (Table S4 and Figure S7). Homologous genes in the MEP, SK, and chlorophyll degradation pathways referred to the description of Almeida *et.al* [84]. The subsequent steps of comparative alignment and *in silico* mapping proceeded as in previous reports [41], [75].

### Cloning and Genetic Mapping of Homologous Genes in the Tocopherol Biosynthesis Pathway

Conservative and specific primers were designed based on putative *A. thaliana* gene sequence information (*VTE1, VTE2, VTE3, VTE4, PDS1*) in NCBI (www.ncbi.nlm.nih.gov) or were based on the *Brassica oleracea* sequence (*VTE5*) in BrassicaDB (http://brassica.bbsrc.ac.uk/BrassicaDB/) to develop probes for bacterial artificial chromosome (BAC) library screening (Table S9). The JnBn BAC library was constructed from the Tapidor variety [85]. Six ^33^P-labeled probes related to *VTE1, VTE2, VTE3, VTE4, VTE5*, and *PDS1* were used to screen this library, resulting in the identification of 151 positive BAC clones. Sequences from the positive BACs for each gene were used for primer design and genetic mapping. For mapping these putative genes in the TNDH population, primer pairs vte4-1f/vte4-1r, vte2-1f/vte2-1r, vte5f/vte5r, pds1-3f/pds1-3r, and vte3-2f/vte3-2r were used to map *BnaA.VTE4.a, BnaA.VTE2.a*, *BnaC.VTE5*, *BnaA.PDS1.c*, and *BnaC.VTE3.b*, respectively (Table S9). Marker nomenclature derived from homologous genes in *B. napus* [86]. The sequences of these homologous genes in *B. napus* were deposited in GenBank under accession numbers JN834015 to JN834026 and EU637012.

### Population Structure and Kinship Evaluation in the Association Panel

The association panel was genotyped with 101 molecular markers, which resulted in 327 polymorphism loci. Polymorphism loci with frequencies below 10% were excluded to avoid the effect of rare alleles; 224 polymorphism loci were identified for use in subsequent analysis.

One hundred and two polymorphism loci, derived from 40 molecular markers evenly distributed on 19 TNDH linkage groups, were used to evaluate population structure (Table S5). These markers were used to test 142 accessions based on the method described by Chen *et al.* [87]. STRUCTURE 2.2, which is based on Bayesian clustering, was implemented for assigning the natural accessions into subpopulations [32]. We tested various numbers of subpopulations ranging from *k* = 1 to *k* = 10. Five runs were processed for each *k* value with 100,000 burn-in length and 100,000 iterations. The results (Q matrix) of replicate runs output from STRUCTURE were integrated by the CLUMPP software [88]. Subsequently, the number of subpopulations were determined by the Δ*k* method [89]. PCA was carried out based on data from the same markers with the software NTYSpc [90]. A covariance matrix exported from NTYSpc was used for subsequent association analysis. The effects of population structure for all traits were evaluated by SAS PROC GLM. The model randomly included two of the three Q matrices on the condition of *k* = 3.

Kinship was estimated with the software SPAGeDi based on data from all 101 markers [38]. All negative kinship coefficients were set to zero and then multiplied twice prior to association analysis [91]. The effects of kinship for all traits were tested with TASSEL V3.0 with MLM model and calculated as *h* ^2^ = *σ^2^_a_*/(*σ^2^_a_*+*σ^2^_e_*), where *σ^2^_a_* and *σ^2^_e_* were the genetic variance and the error variance, respectively [92].

### Linkage Disequilibrium Evaluation

LD between markers was evaluated with TASSEL V3.0 by calculating *r* ^2^ between makers. Loci on the same linkage group were used to evaluate LD decay. The threshold of significant LD for these linked loci was defined as the 95% quantile of the *r* ^2^ value among unlinked loci pairs. LD decay with genetic distance was tested by nonlinear regression of *r* ^2^ values [39], [93].

### Model Comparisons and Association Analysis

Six models were compared to choose the most suitable model for each trait. The first model, ANOVA, did not consider population structure (Q), PCA, or kinship (K) effects. The second model (Q) considered Q effects, while the third model, PCA, considered the population structure effects developed from PCA. The fourth model (K) considered kinship effects, while the fifth model (PCA+K) considered both PCA and K effects and the last model (Q+K) considered both Q and K effects. The ANOVA, Q, and PCA model were calculated by GLM in TASSEL V3.0, while the K, PCA+K, and Q+K models were evaluated by MLM in TASSEL V3.0 [92]. The quantile-quantile plots of estimated –log10 (*p*) were constructed from the observed *p* values from the marker-phenotype association and the expected *p* value, supposing that no associations were observed between markers and traits [94]. Finally, association analysis was carried out with the best suitable model for each trait with TASSEL V3.0.

## Supporting Information

Figure S1 **Phenotypic variation between two parents of TNDH in three environments.** The following variations are depicted: (A) α-tocopherol content, (B) γ-tocopherol content, (C) total tocopherol content, (D), tocopherol composition.
(TIF)

Figure S2 **Distribution of tocopherol content and composition in TNDH, RC−F_2_ populations and the association panel.**
(PPT)

Figure S3 **Distribution of 57 QTL on TNDH linkage groups.** α, α-tocopherol content; γ, γ-tocopherol content; t, total tocopherol content; α/γ, tocopherol composition.
(TIF)

Figure S4 **Schematic of the chromosomal components of the nearly isogenic lines by molecular markers in** ***qTOC.A7*** **and** ***qTOC.A9*** **.**
(PPT)

Figure S5 **Genetic mapping of BnaA.VTE2.a, BnaC.VTE3.b, BnaA.VTE4.a, BnaC.VTE5, and BnaA.PDS1.c.**
(XLS)

Figure S6 **Plot of linkage disequilibrium (LD) extent (** ***r*** **^2^) against genetic map distance (cM) on A9.** (a) Overview of LD decay on A9. (b) An enhanced view of LD decay on the whole genome. Blue solid line, nonlinear regression trend line of *r^2^* against the genetic map distance. Black dashed line, threshold as the 95% quantile of the *r* ^2^ value among unlinked loci pairs.
(TIF)

Figure S7 **Comparative alignment between** ***Brassica napus*** **and** ***Arabidopsis thaliana*** **.**
(XLS)

Table S1 **QTL detected in the TNDH and RC−F_2_ populations.**
(XLS)

Table S2 **Nonredundant QTL for tocopherol content and composition in the TNDH and RC−F_2_ populations.**
(XLS)

Table S3 **Epistatic interacting loci pairs in the TNDH and RC-F_2_ populations.**
(XLS)

Table S4 **TNDHlinkage map and** ***in silico*** **mapping results.**
(XLS)

Table S5 **Natural varieties and molecular markers for population evaluation and association analysis.**
(XLS)

Table S6 **Subpopulations divided by Structure and principal component analysis.**
(XLS)

Table S7 **Associated markers for tocopherol content and composition in the association panel.**
(XLS)

Table S8 **Comparison of QTL mapping results between the TNDH and the MSDH population.**
(XLS)

Table S9 **Primers for probes and homologous gene mapping.**
(XLS)
