# Supplementary figures and images for: Unraveling the Genetic Basis of Seed Tocopherol Content and Composition in Rapeseed (Brassica napus L.)
Source: PLoS One. 2012 Nov 20;7(11):e50038. doi: 10.1371/journal.pone.0050038 (PMC3502226; doi:10.1371/journal.pone.0050038)

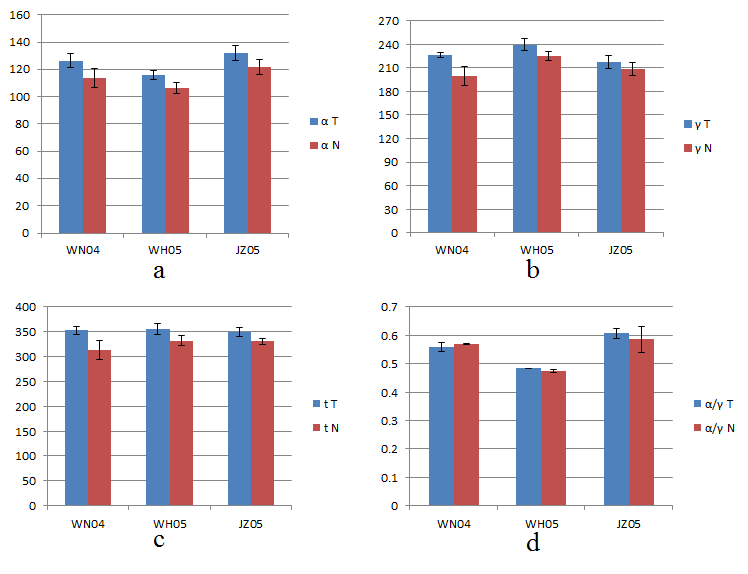

Supplement: Figure S1 — Phenotypic variation between two parents of TNDH in three environments. The following variations are depicted: (A) α-tocopherol content, (B) γ-tocopherol content, (C) total tocopherol content, (D), tocopherol composition. (TIF) [file pone.0050038.s001.tif]

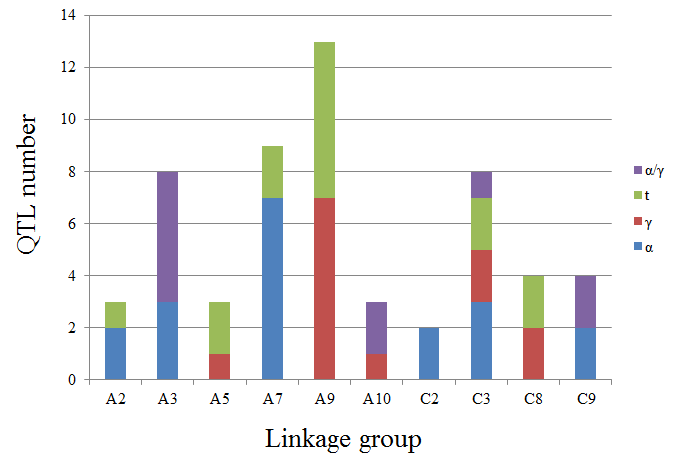

Supplement: Figure S3 — Distribution of 57 QTL on TNDH linkage groups. α, α-tocopherol content; γ, γ-tocopherol content; t, total tocopherol content; α/γ, tocopherol composition. (TIF) [file pone.0050038.s003.tif]

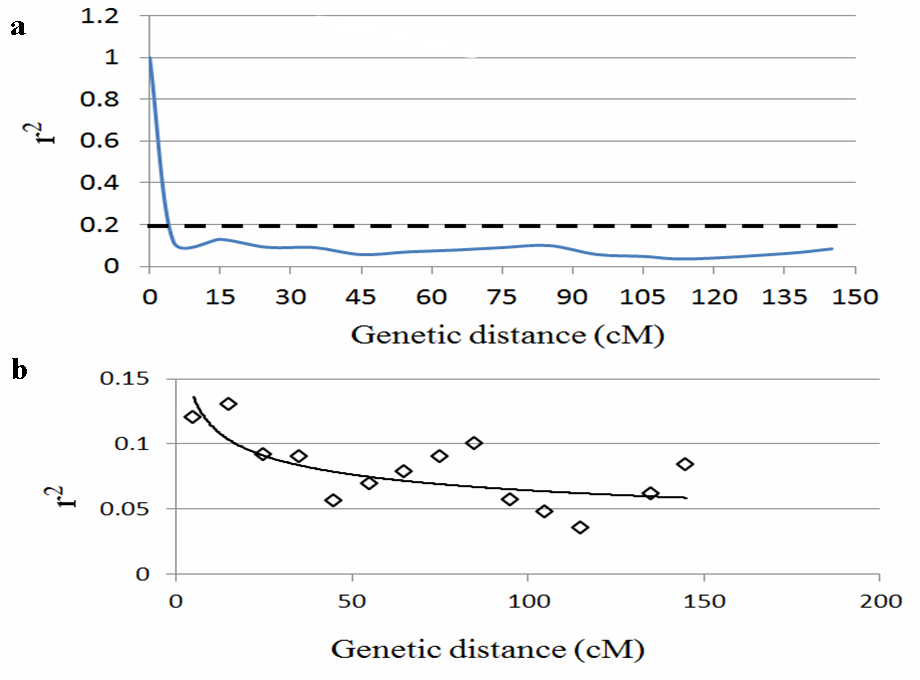

Supplement: Figure S6 — Plot of linkage disequilibrium (LD) extent ( r 2) against genetic map distance (cM) on A9. (a) Overview of LD decay on A9. (b) An enhanced view of LD decay on the whole genome. Blue solid line, nonlinear regression trend line of r2 against the genetic map distance. Black dashed line, threshold as the 95% quantile of the r 2 value among unlinked loci pairs. (TIF) [file pone.0050038.s006.tif]
