# Supplementary material for: Unraveling the Genetic Basis of Seed Tocopherol Content and Composition in Rapeseed (Brassica napus L.)
Source: PLoS One. 2012 Nov 20;7(11):e50038. doi: 10.1371/journal.pone.0050038 (PMC3502226; doi:10.1371/journal.pone.0050038)
Supplement: Figure S2 — Distribution of tocopherol content and composition in TNDH, RC−F2 populations and the association panel. (PPT) [file pone.0050038.s002.ppt]

## Slide 1
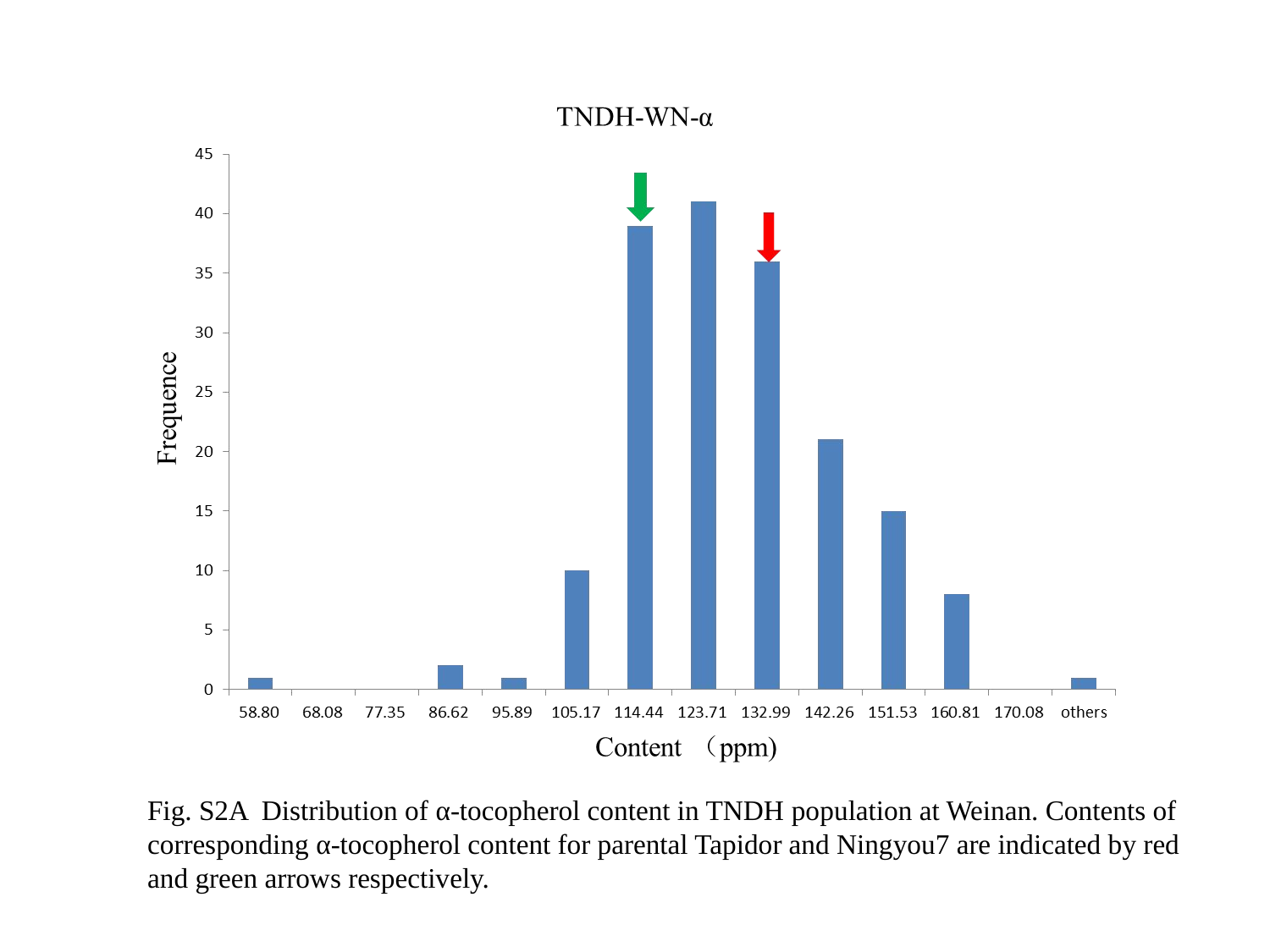

Fig. S2A Distribution of α-tocopherol content in TNDH population at Weinan. Contents of corresponding α-tocopherol content for parental Tapidor and Ningyou7 are indicated by red and green arrows respectively.

## Slide 2
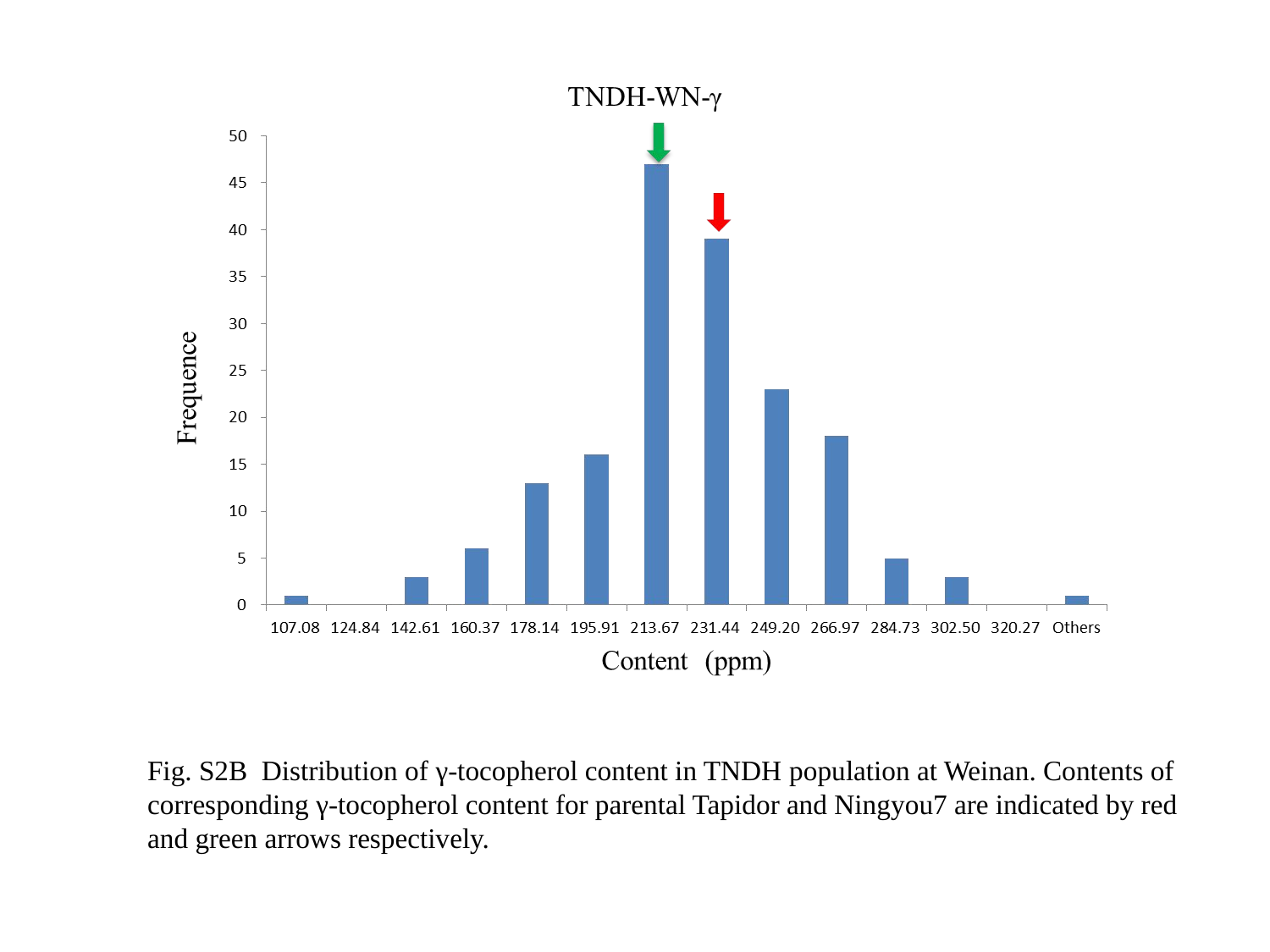

Fig. S2B Distribution of γ-tocopherol content in TNDH population at Weinan. Contents of corresponding γ-tocopherol content for parental Tapidor and Ningyou7 are indicated by red and green arrows respectively.

## Slide 3
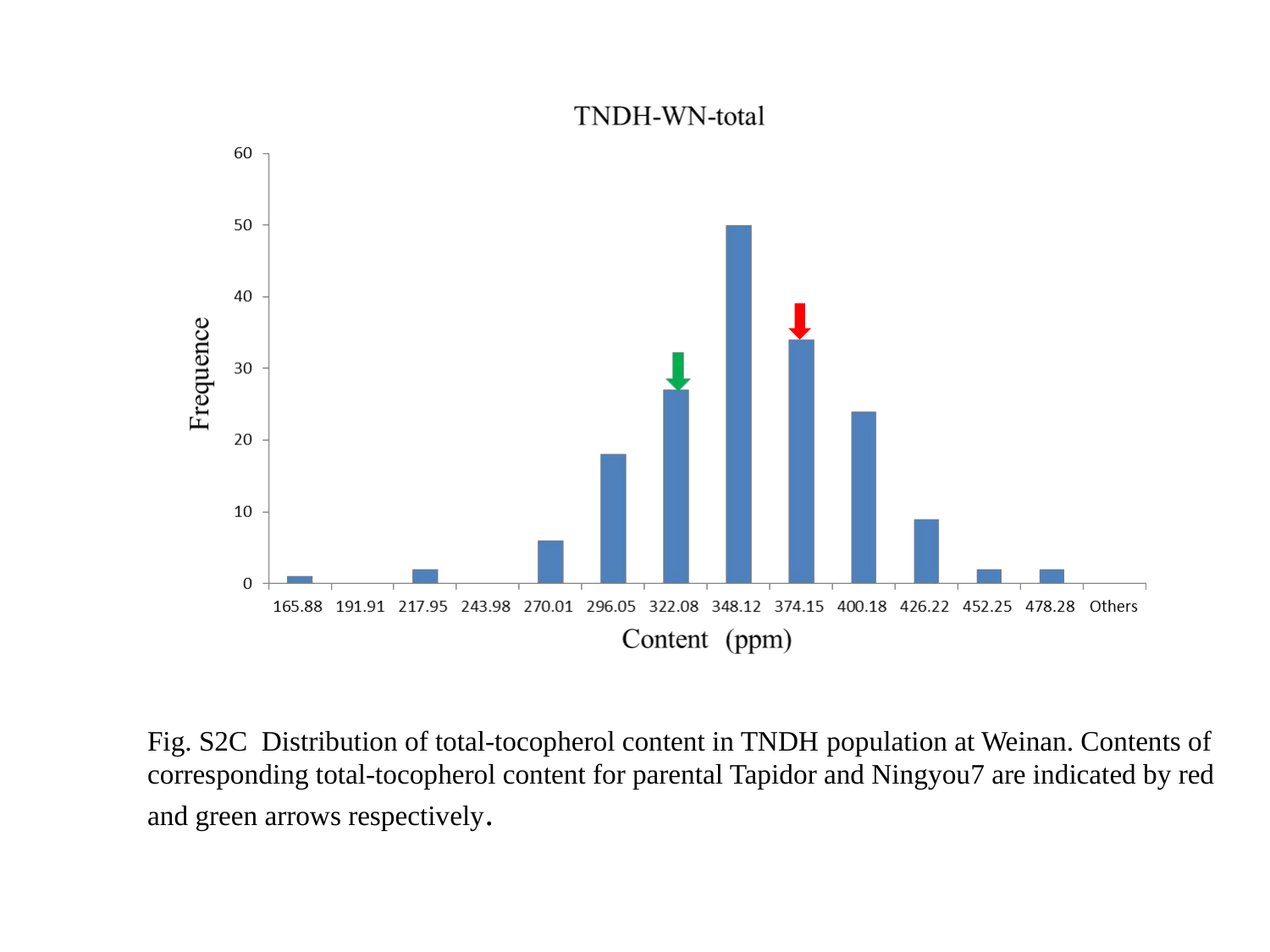

Fig. S2C Distribution of total-tocopherol content in TNDH population at Weinan. Contents of corresponding total-tocopherol content for parental Tapidor and Ningyou7 are indicated by red and green arrows respectively.

## Slide 4
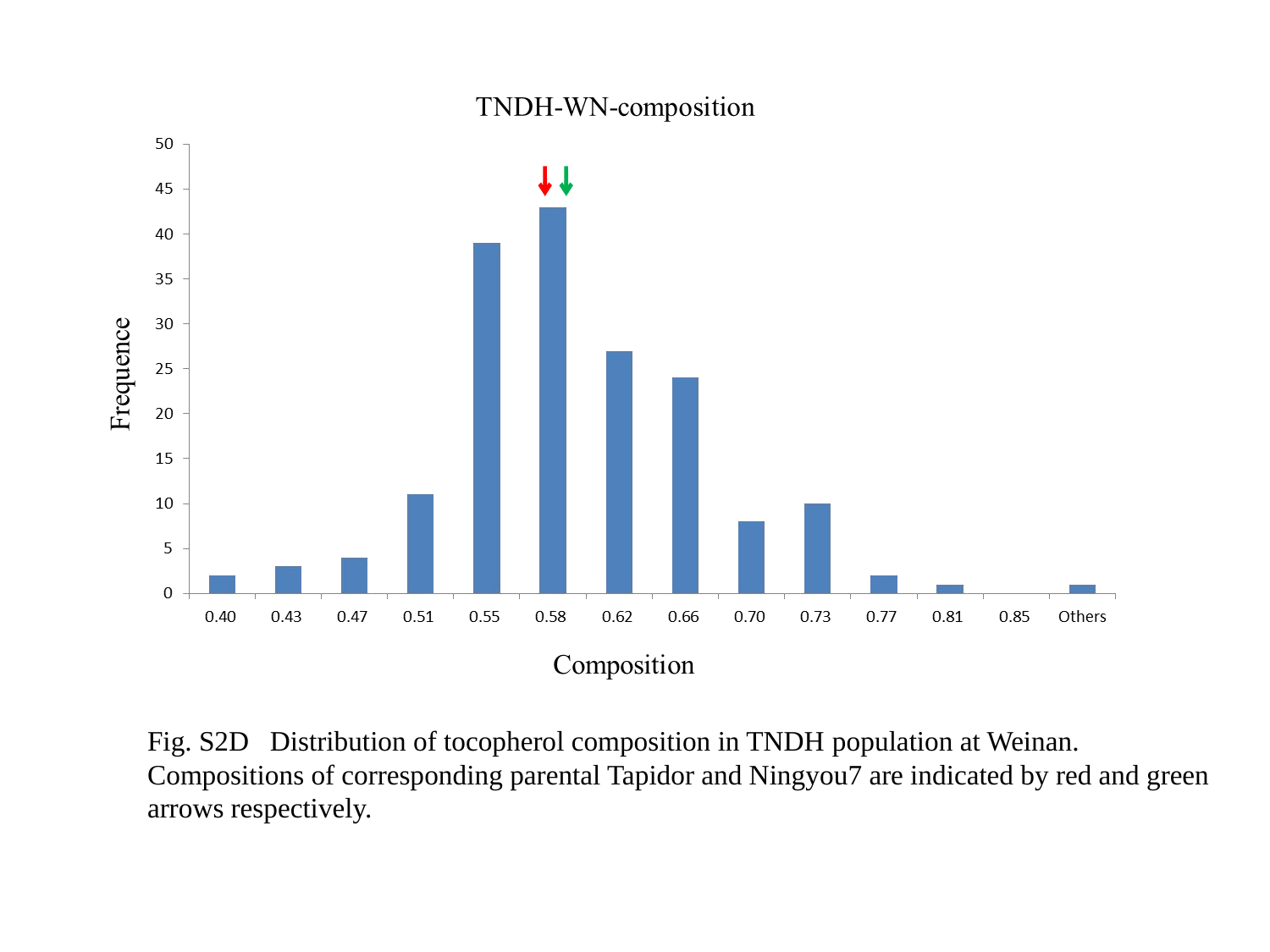

Fig. S2D Distribution of tocopherol composition in TNDH population at Weinan. Compositions of corresponding parental Tapidor and Ningyou7 are indicated by red and green arrows respectively.

## Slide 5
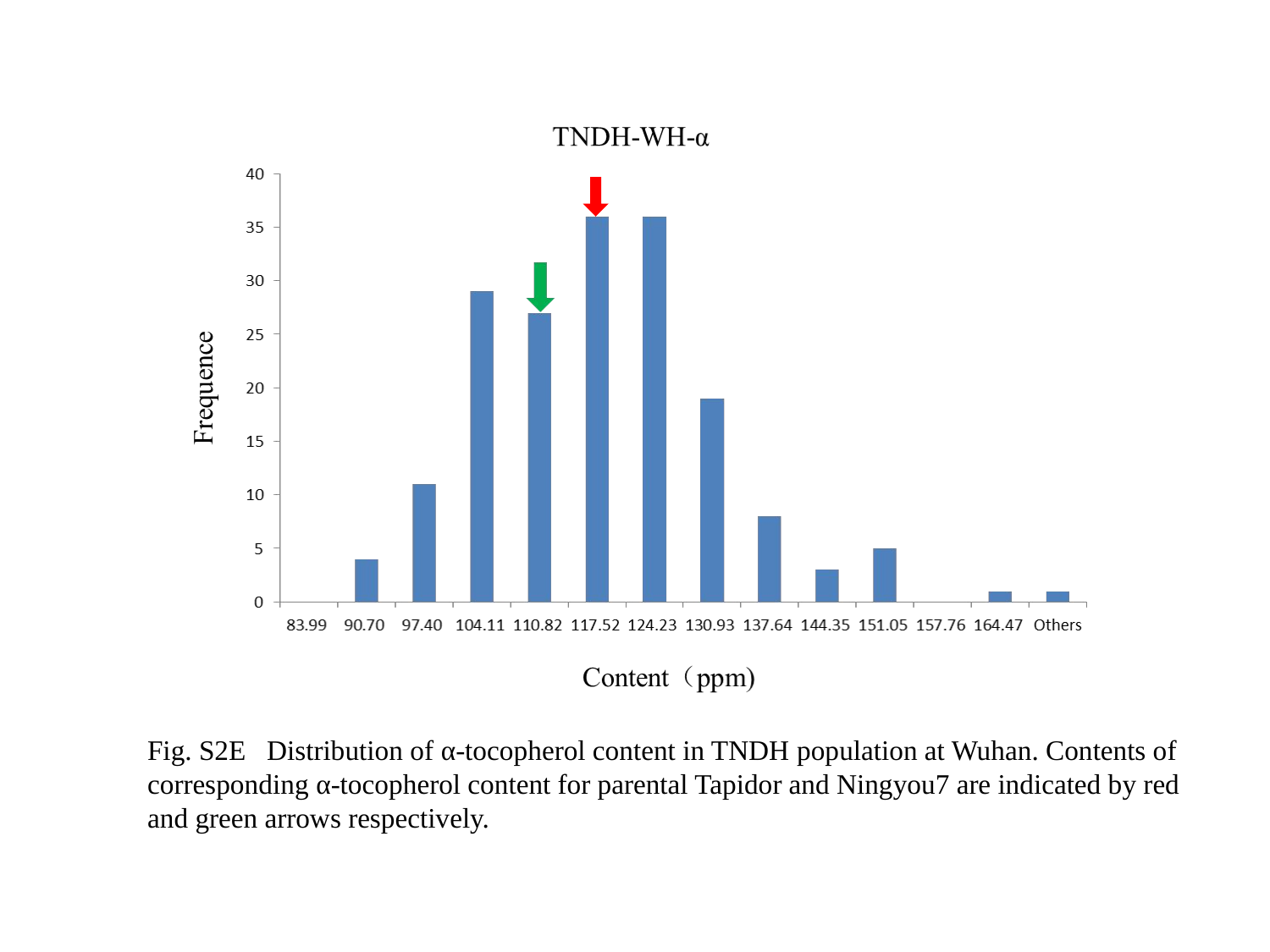

Fig. S2E Distribution of α-tocopherol content in TNDH population at Wuhan. Contents of corresponding α-tocopherol content for parental Tapidor and Ningyou7 are indicated by red and green arrows respectively.

## Slide 6
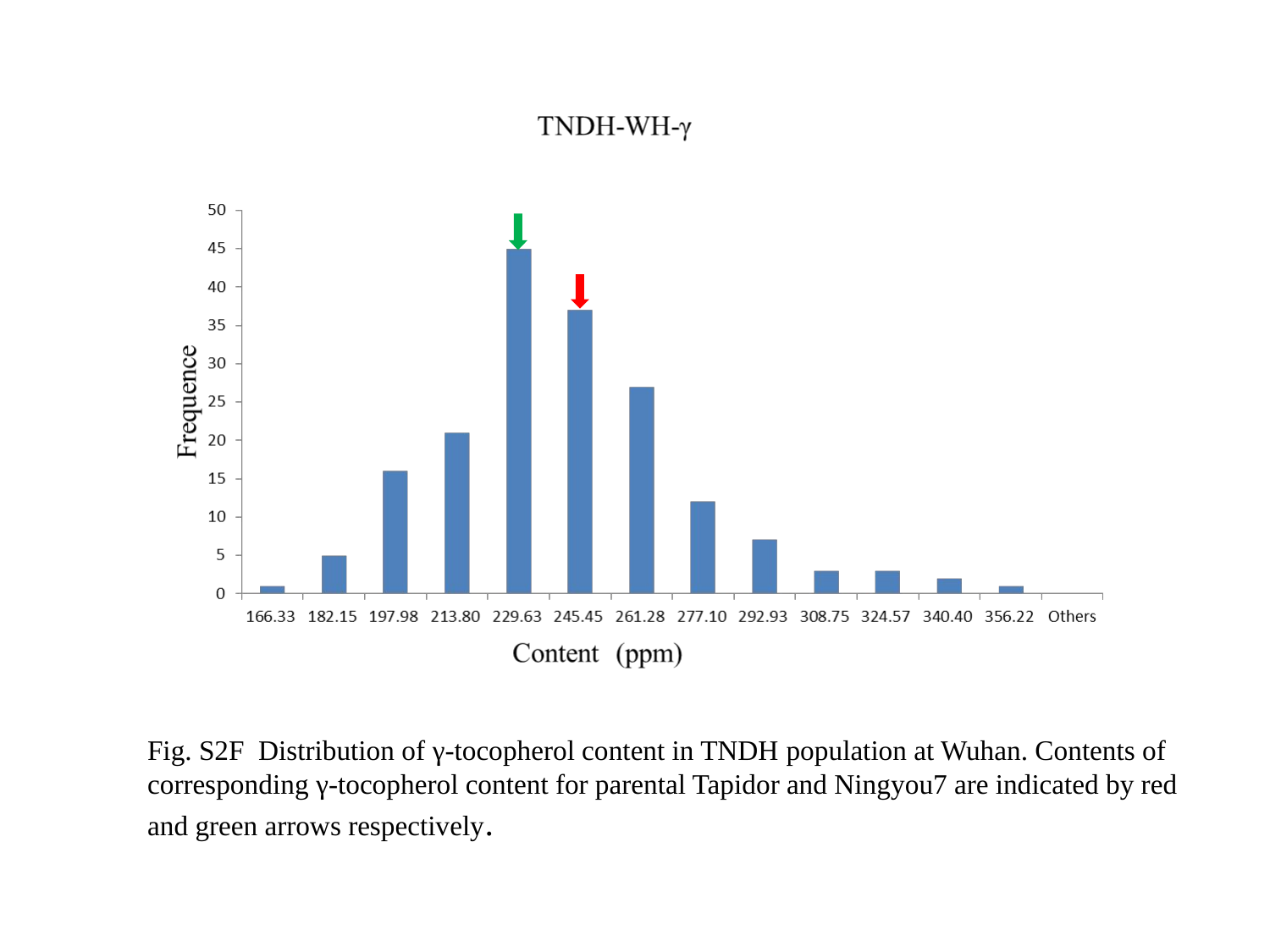

Fig. S2F Distribution of γ-tocopherol content in TNDH population at Wuhan. Contents of corresponding γ-tocopherol content for parental Tapidor and Ningyou7 are indicated by red and green arrows respectively.

## Slide 7
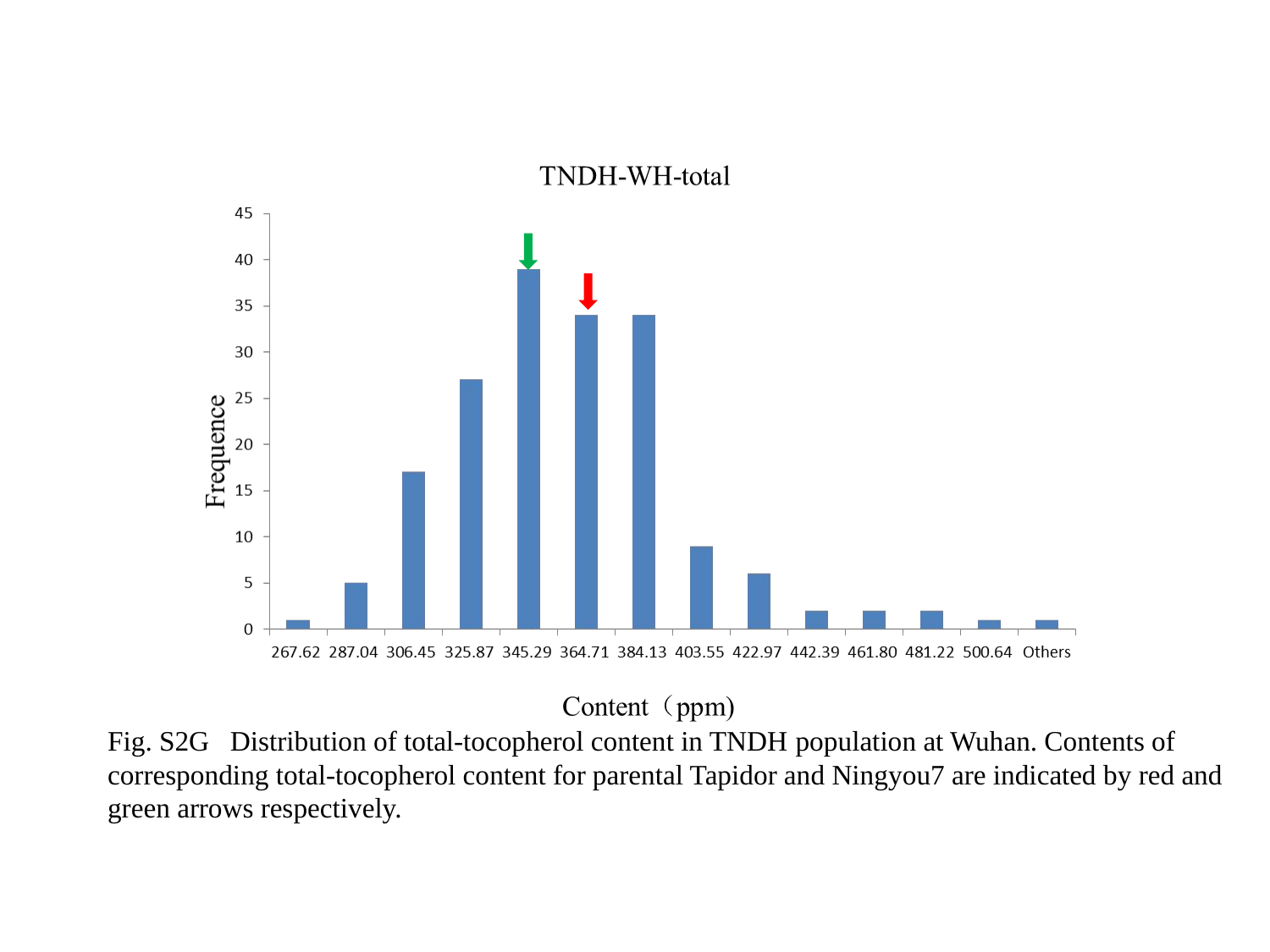

Fig. S2G Distribution of total-tocopherol content in TNDH population at Wuhan. Contents of corresponding total-tocopherol content for parental Tapidor and Ningyou7 are indicated by red and green arrows respectively.

## Slide 8
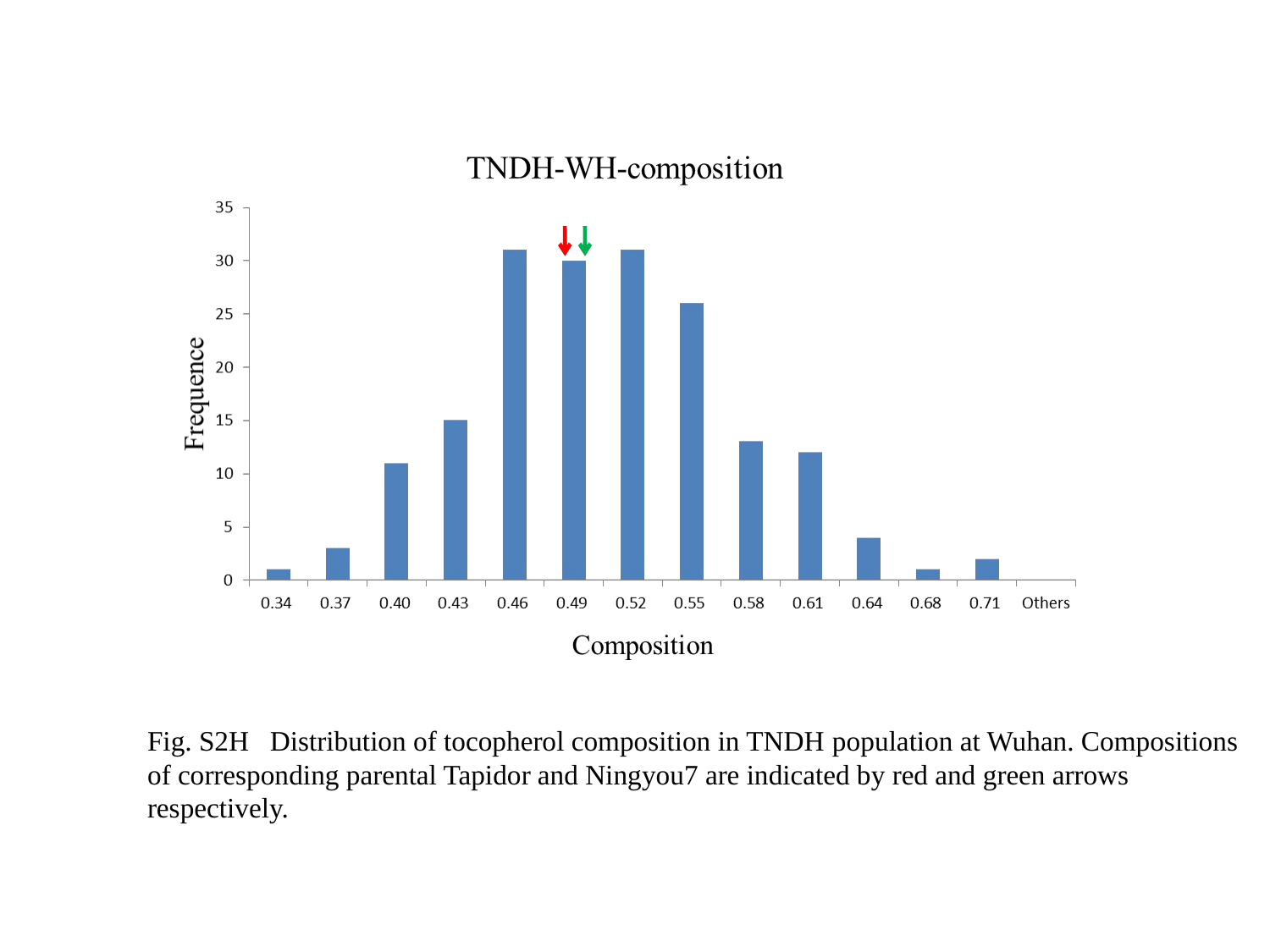

Fig. S2H Distribution of tocopherol composition in TNDH population at Wuhan. Compositions of corresponding parental Tapidor and Ningyou7 are indicated by red and green arrows respectively.

## Slide 9
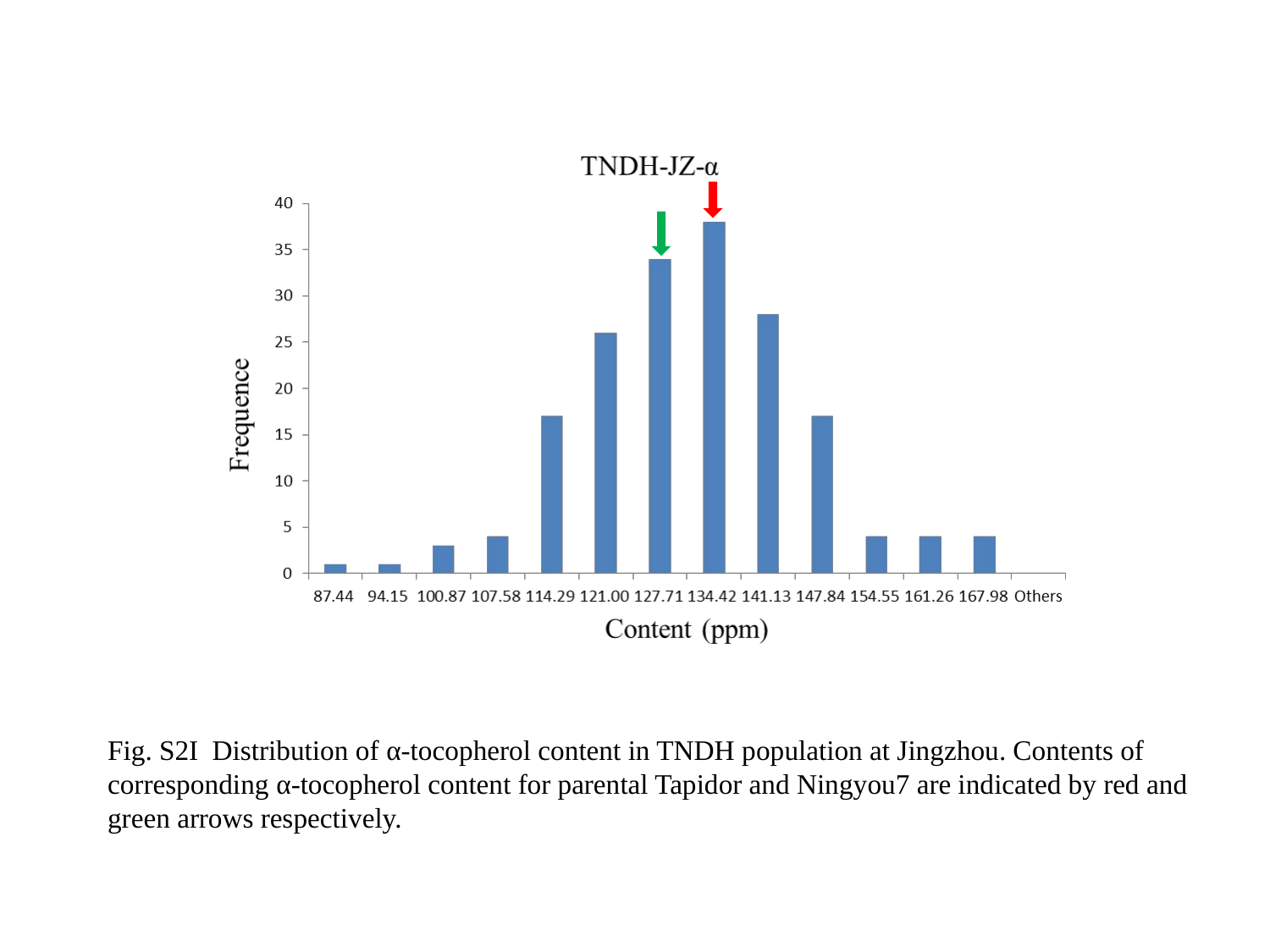

Fig. S2I Distribution of α-tocopherol content in TNDH population at Jingzhou. Contents of corresponding α-tocopherol content for parental Tapidor and Ningyou7 are indicated by red and green arrows respectively.

## Slide 10
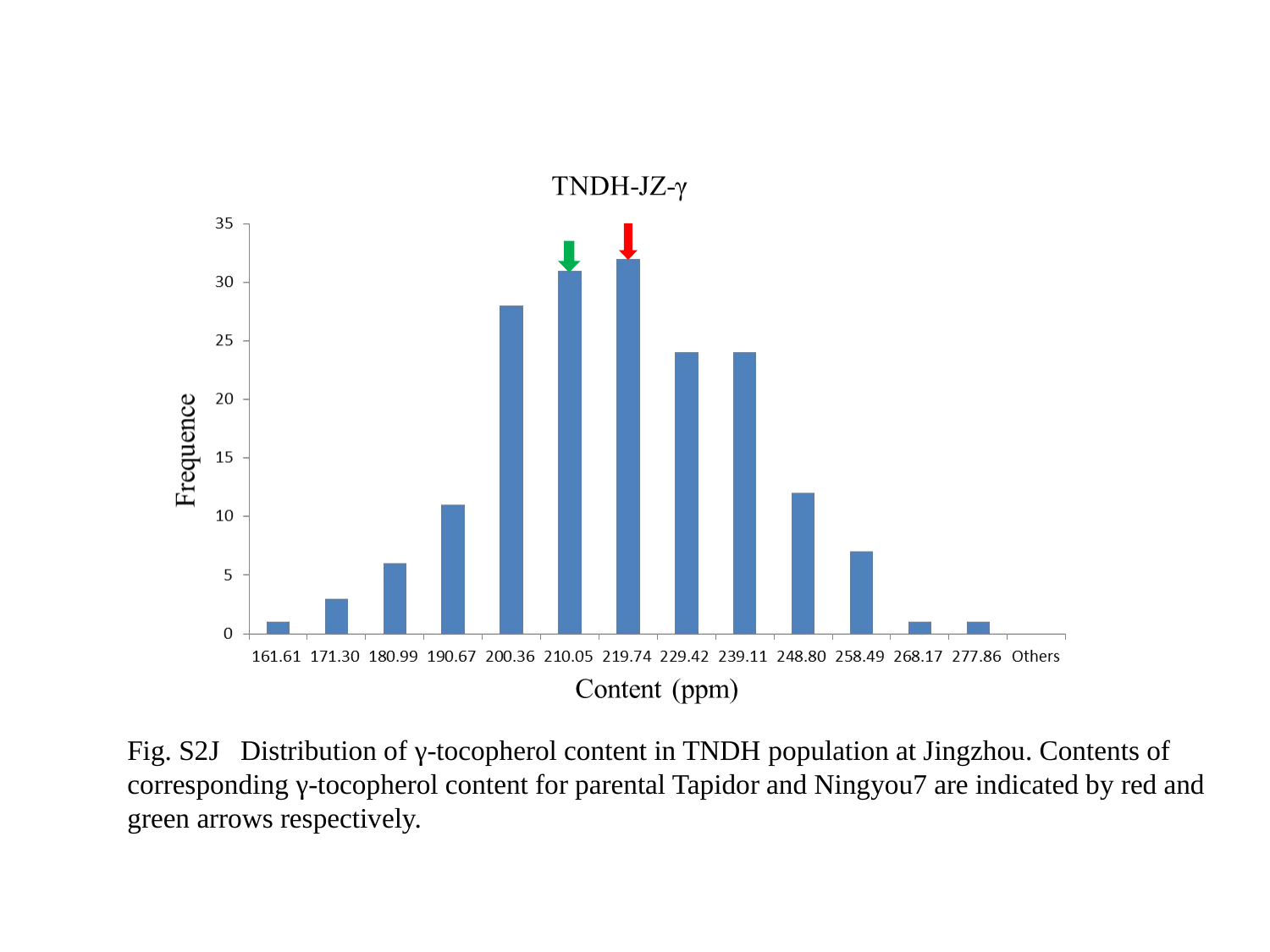

Fig. S2J Distribution of γ-tocopherol content in TNDH population at Jingzhou. Contents of corresponding γ-tocopherol content for parental Tapidor and Ningyou7 are indicated by red and green arrows respectively.

## Slide 11
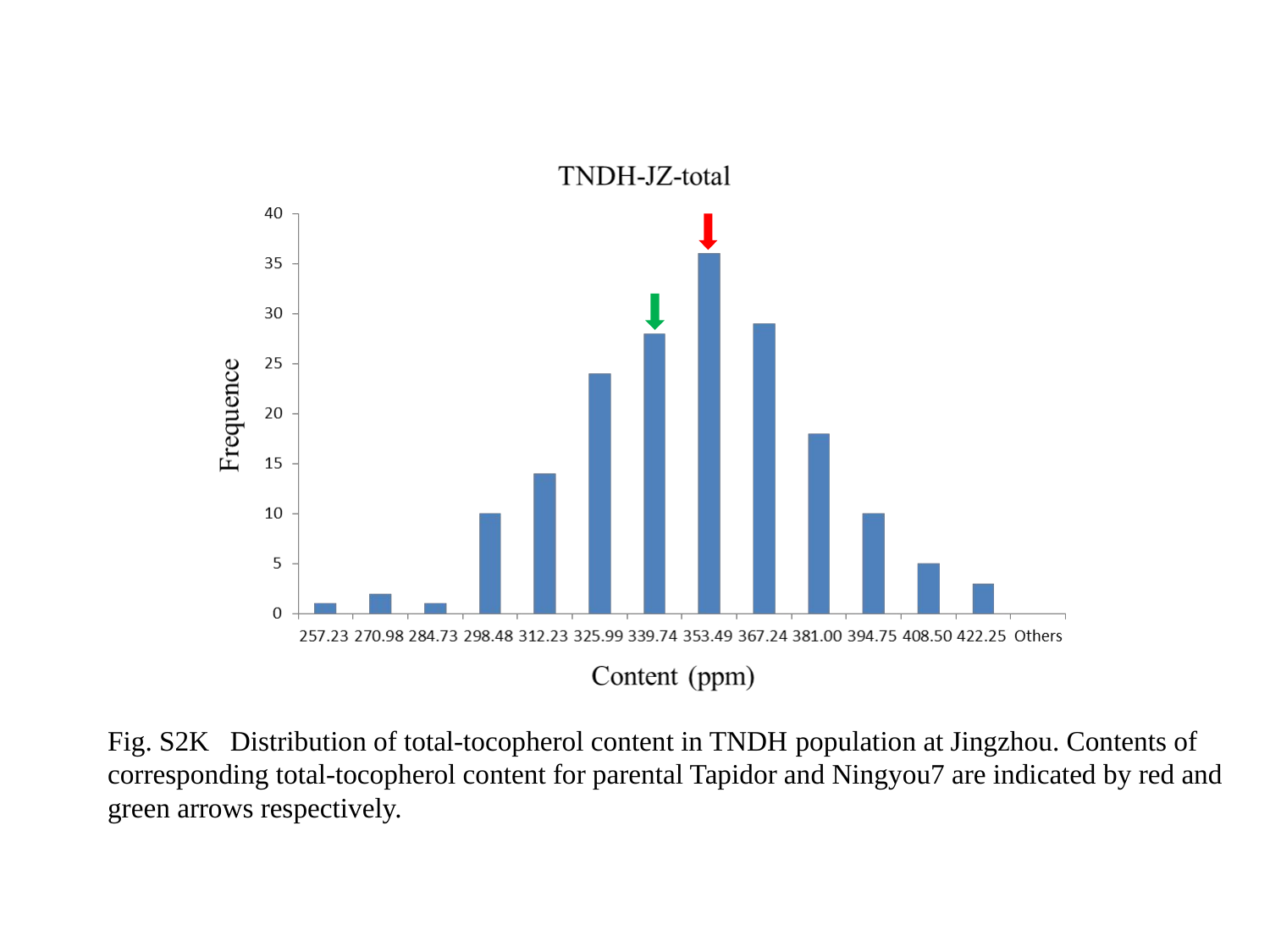

Fig. S2K Distribution of total-tocopherol content in TNDH population at Jingzhou. Contents of corresponding total-tocopherol content for parental Tapidor and Ningyou7 are indicated by red and green arrows respectively.

## Slide 12
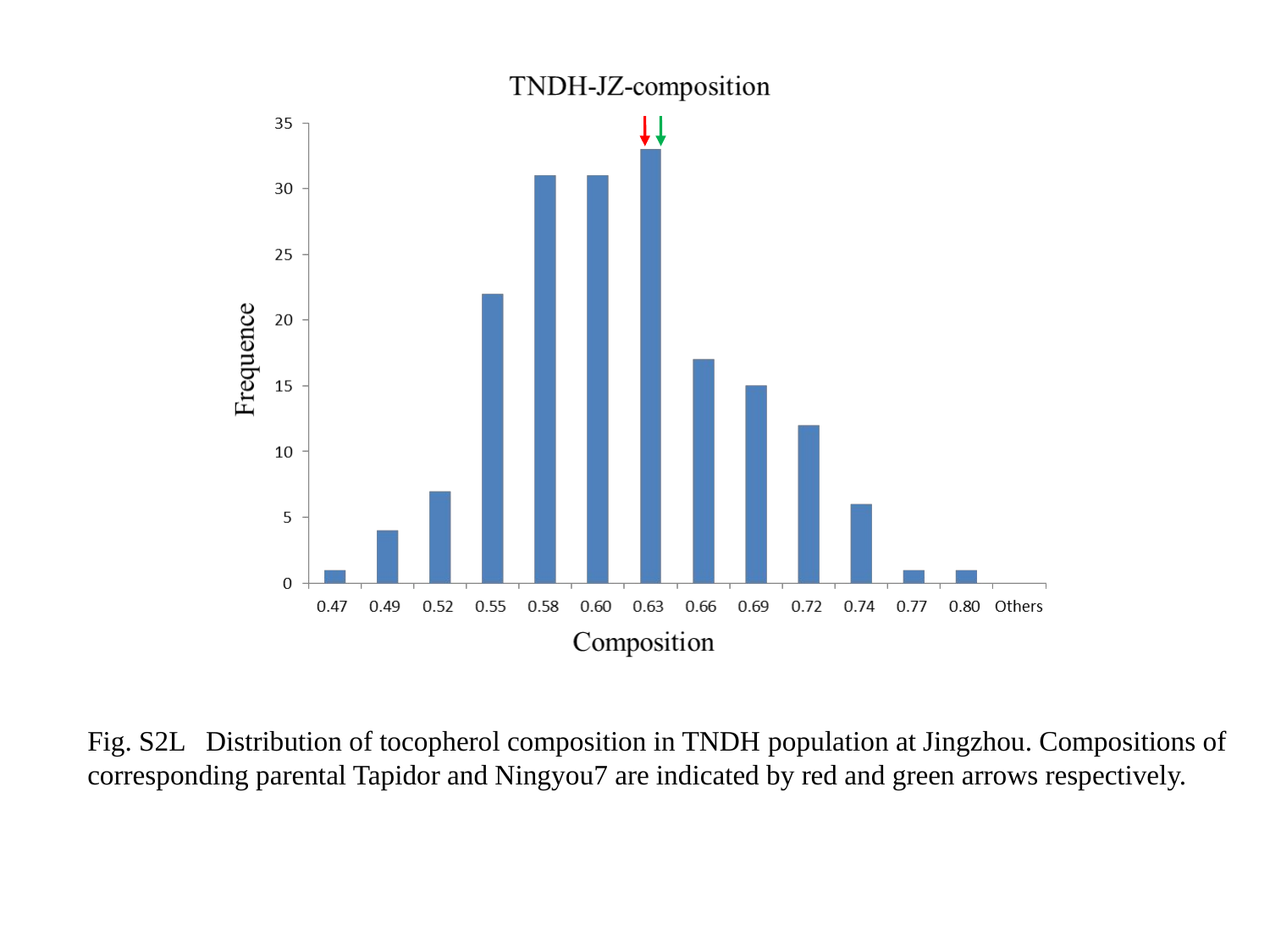

Fig. S2L Distribution of tocopherol composition in TNDH population at Jingzhou. Compositions of corresponding parental Tapidor and Ningyou7 are indicated by red and green arrows respectively.

## Slide 13
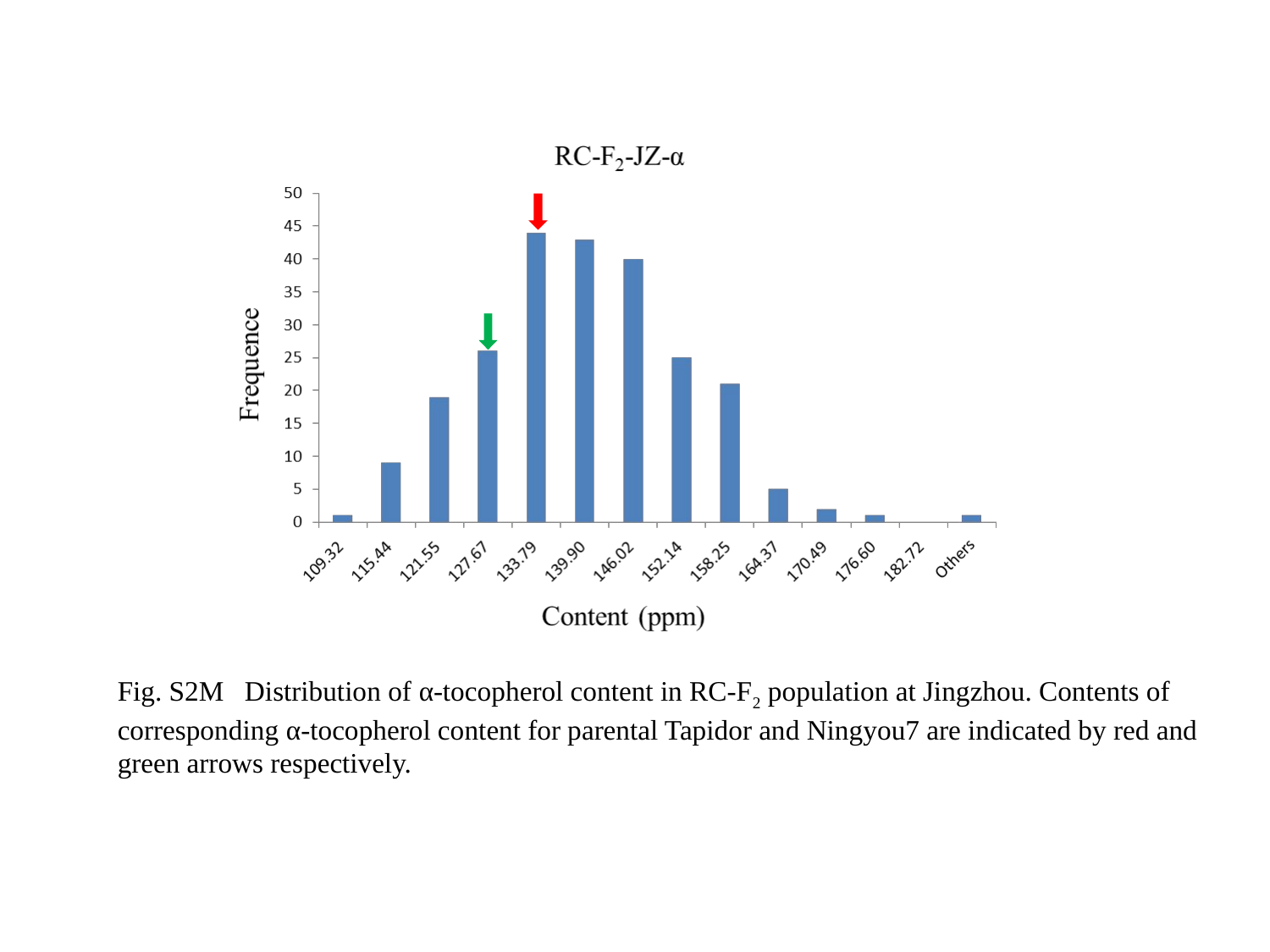

Fig. S2M Distribution of α-tocopherol content in RC-F2 population at Jingzhou. Contents of corresponding α-tocopherol content for parental Tapidor and Ningyou7 are indicated by red and green arrows respectively.

## Slide 14
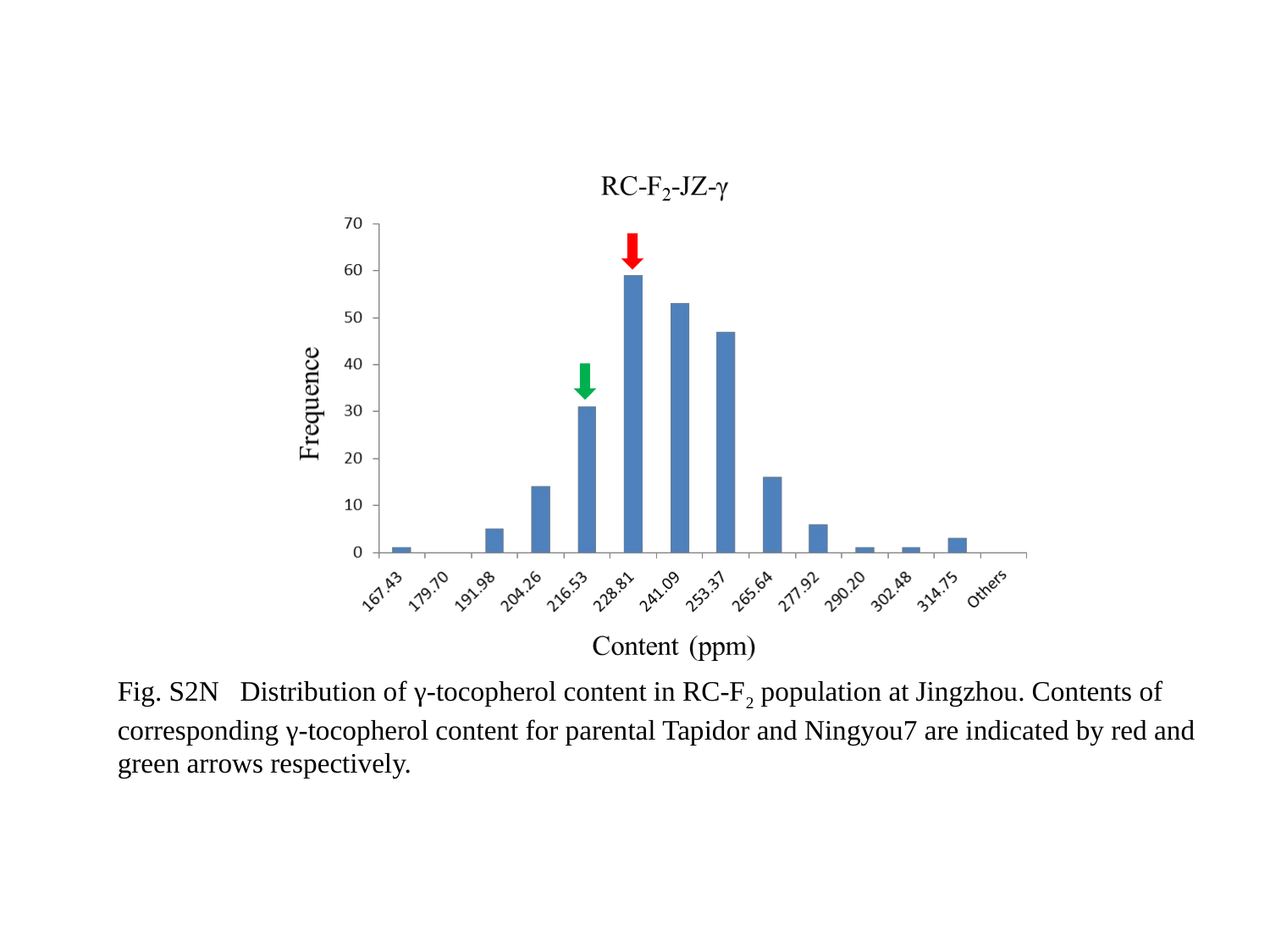

Fig. S2N Distribution of γ-tocopherol content in RC-F2 population at Jingzhou. Contents of corresponding γ-tocopherol content for parental Tapidor and Ningyou7 are indicated by red and green arrows respectively.

## Slide 15
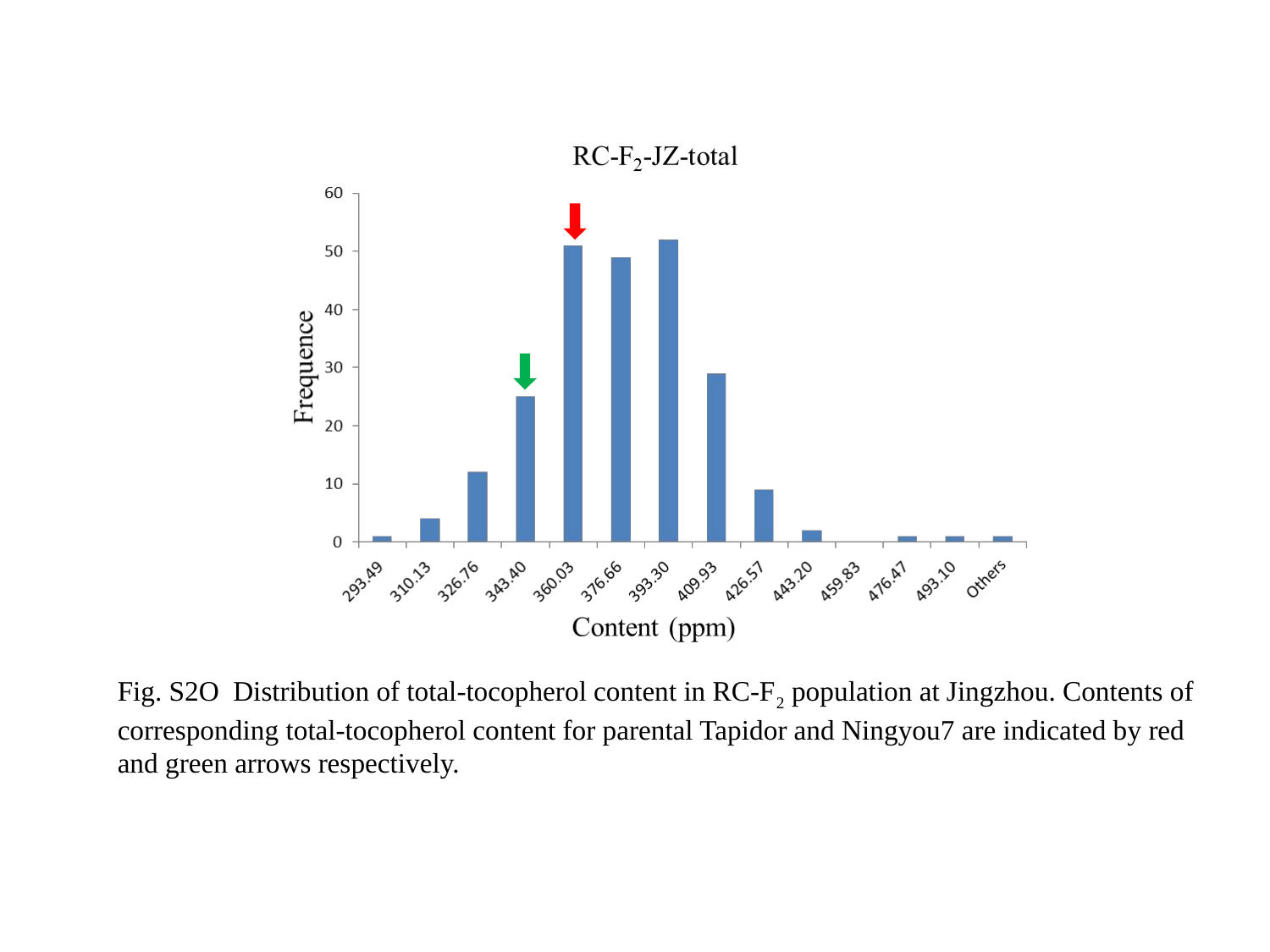

Fig. S2O Distribution of total-tocopherol content in RC-F2 population at Jingzhou. Contents of corresponding total-tocopherol content for parental Tapidor and Ningyou7 are indicated by red and green arrows respectively.

## Slide 16
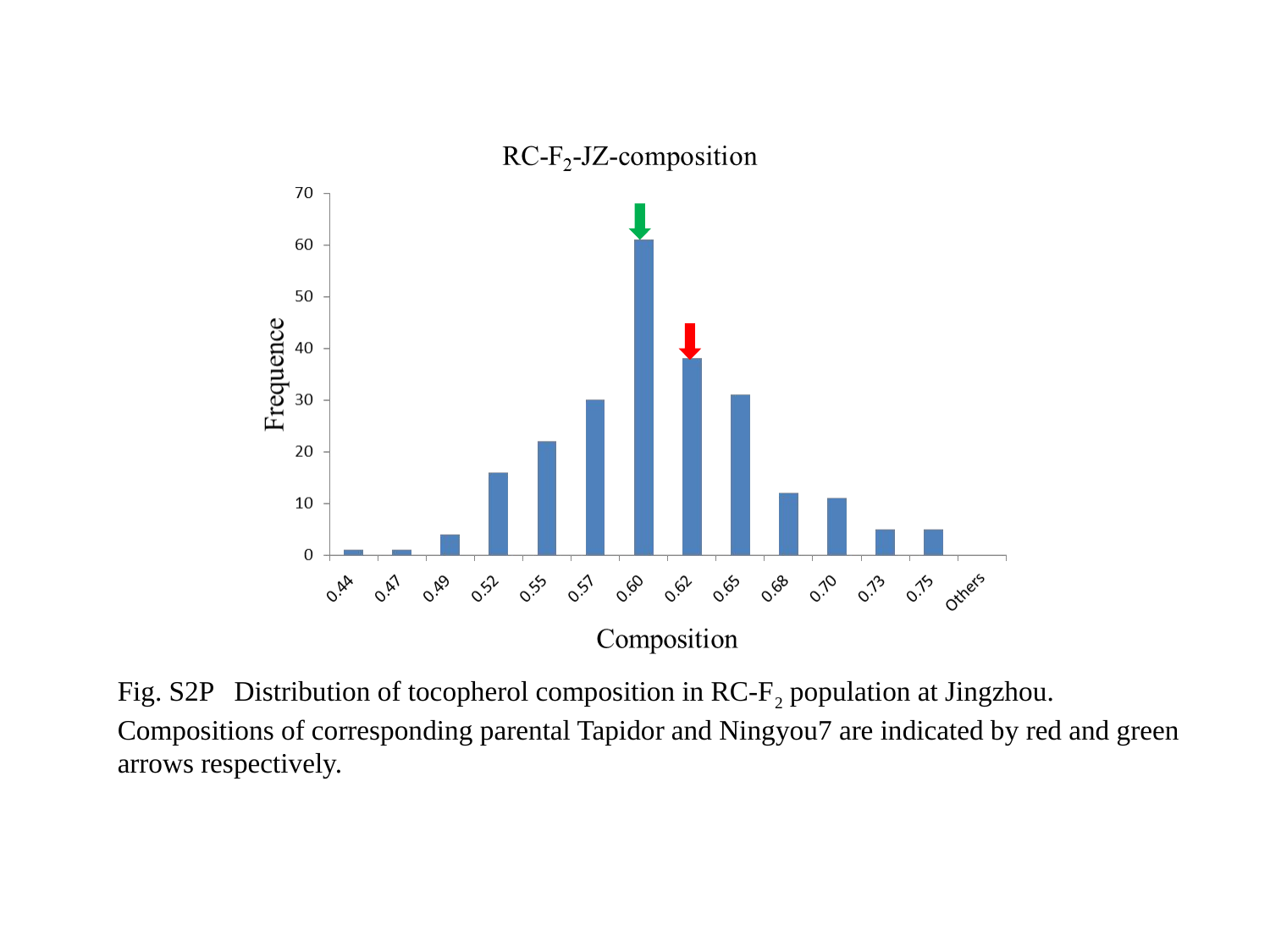

Fig. S2P Distribution of tocopherol composition in RC-F2 population at Jingzhou. Compositions of corresponding parental Tapidor and Ningyou7 are indicated by red and green arrows respectively.

## Slide 17
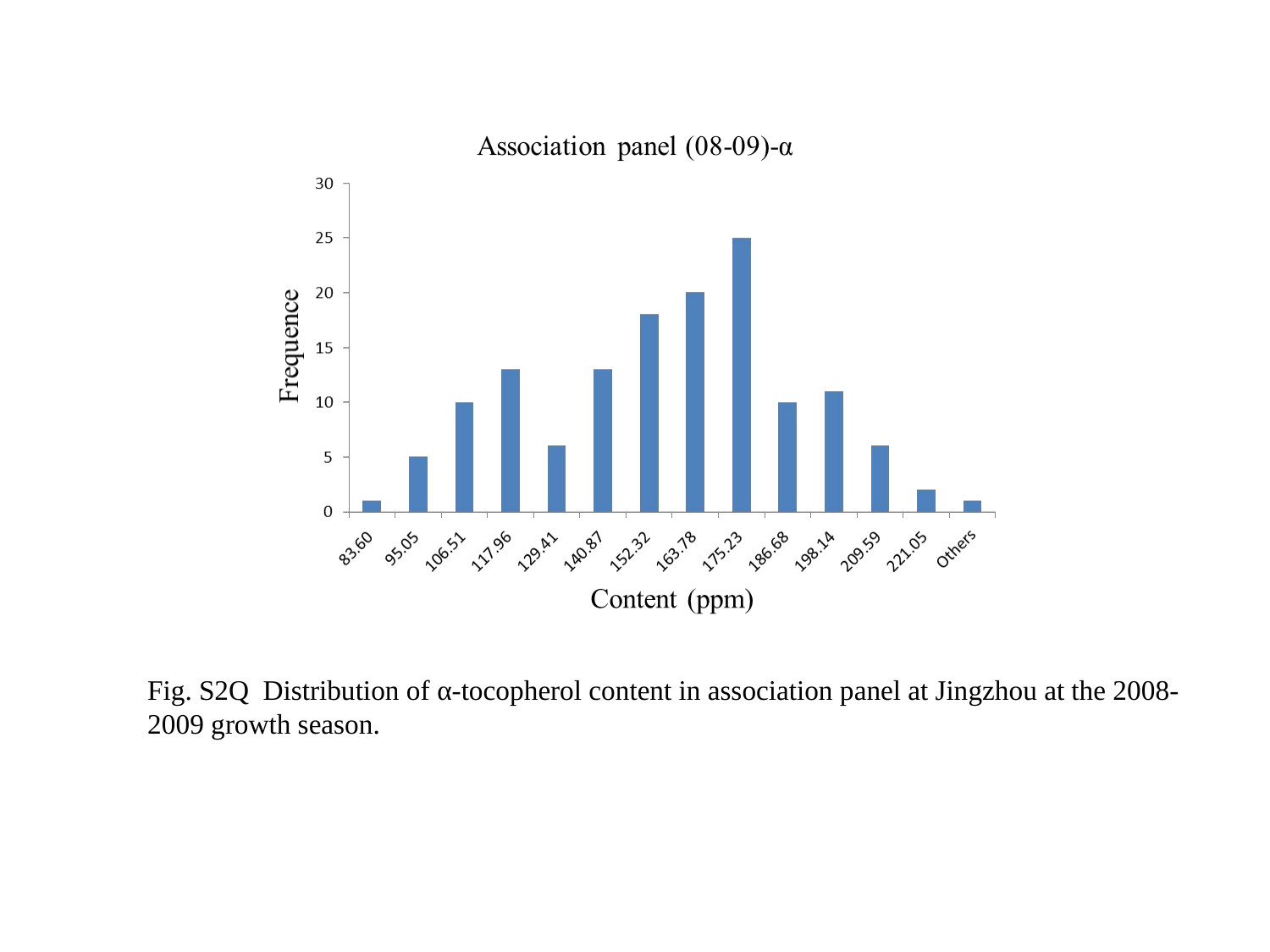

Fig. S2Q Distribution of α-tocopherol content in association panel at Jingzhou at the 2008-2009 growth season.

## Slide 18
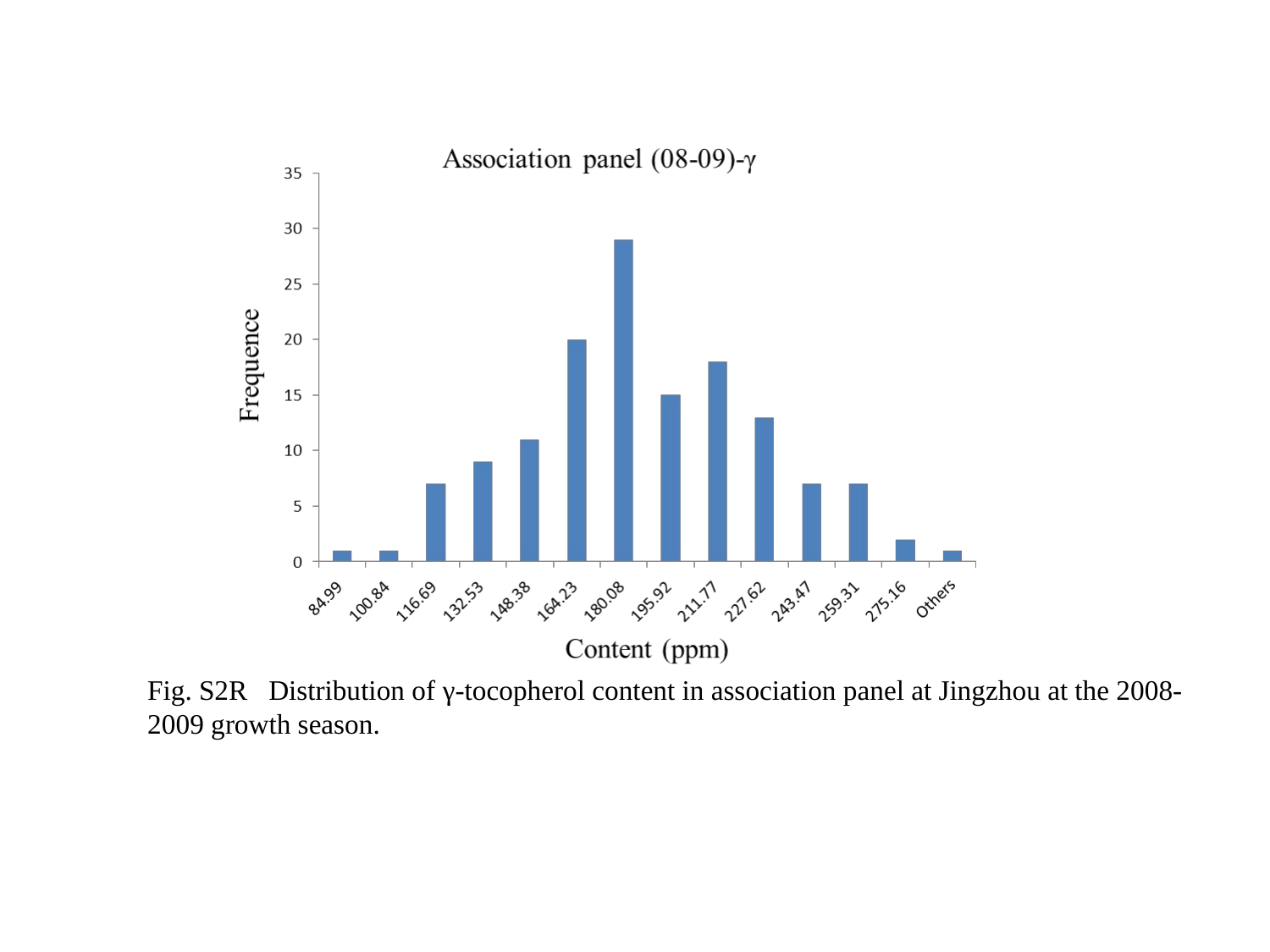

Fig. S2R Distribution of γ-tocopherol content in association panel at Jingzhou at the 2008-2009 growth season.

## Slide 19
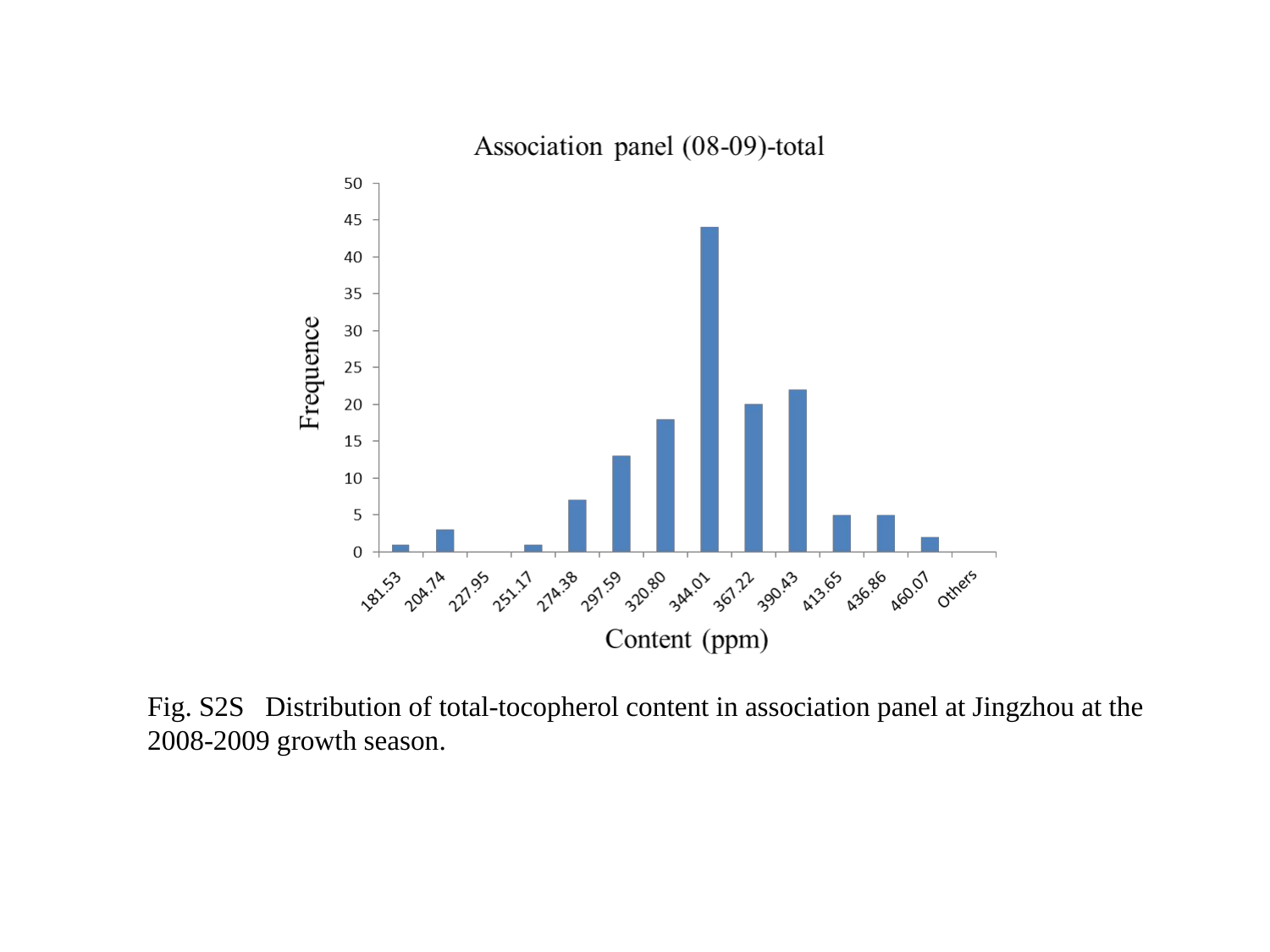

Fig. S2S Distribution of total-tocopherol content in association panel at Jingzhou at the 2008-2009 growth season.

## Slide 20
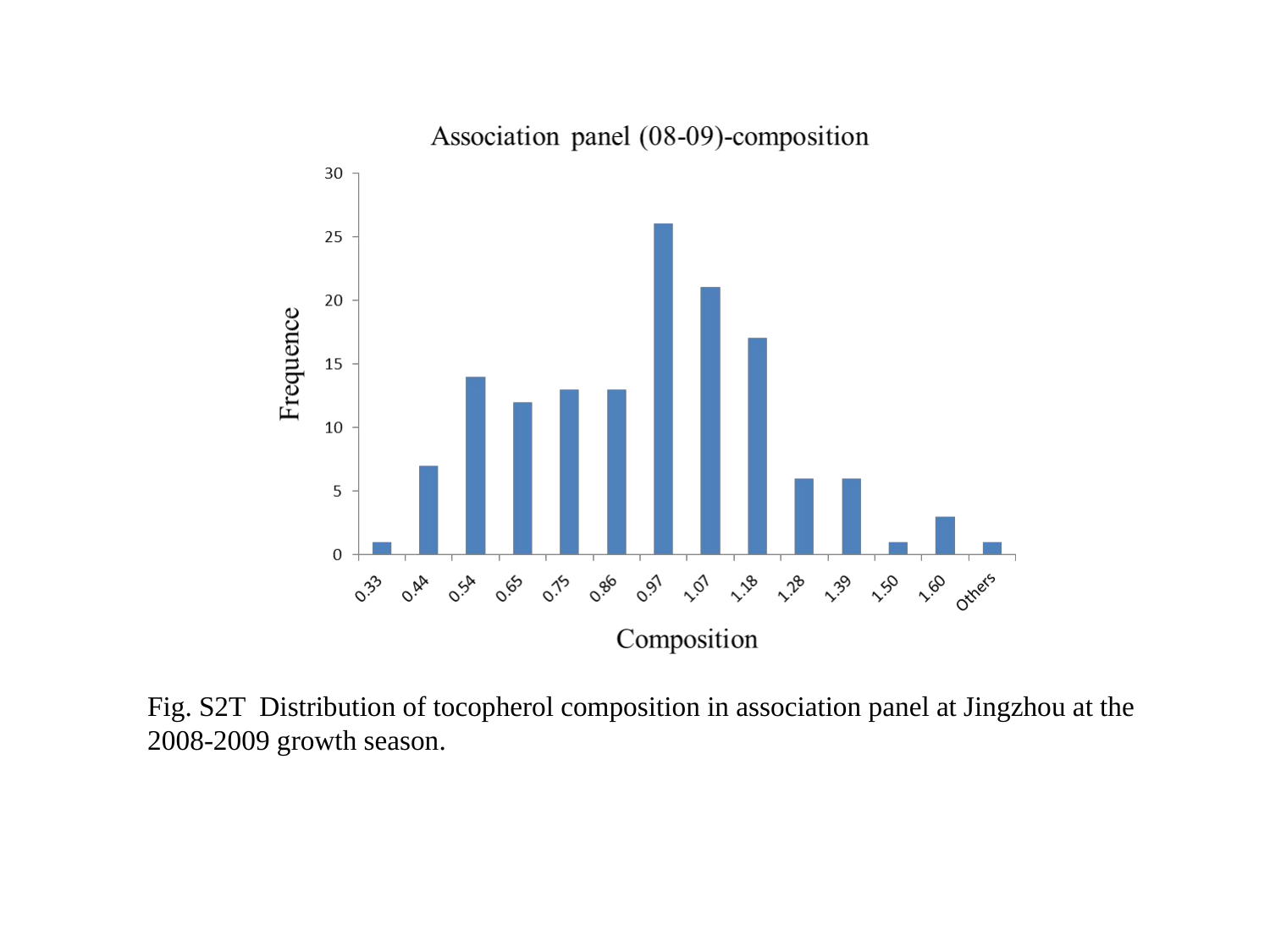

Fig. S2T Distribution of tocopherol composition in association panel at Jingzhou at the 2008-2009 growth season.

## Slide 21
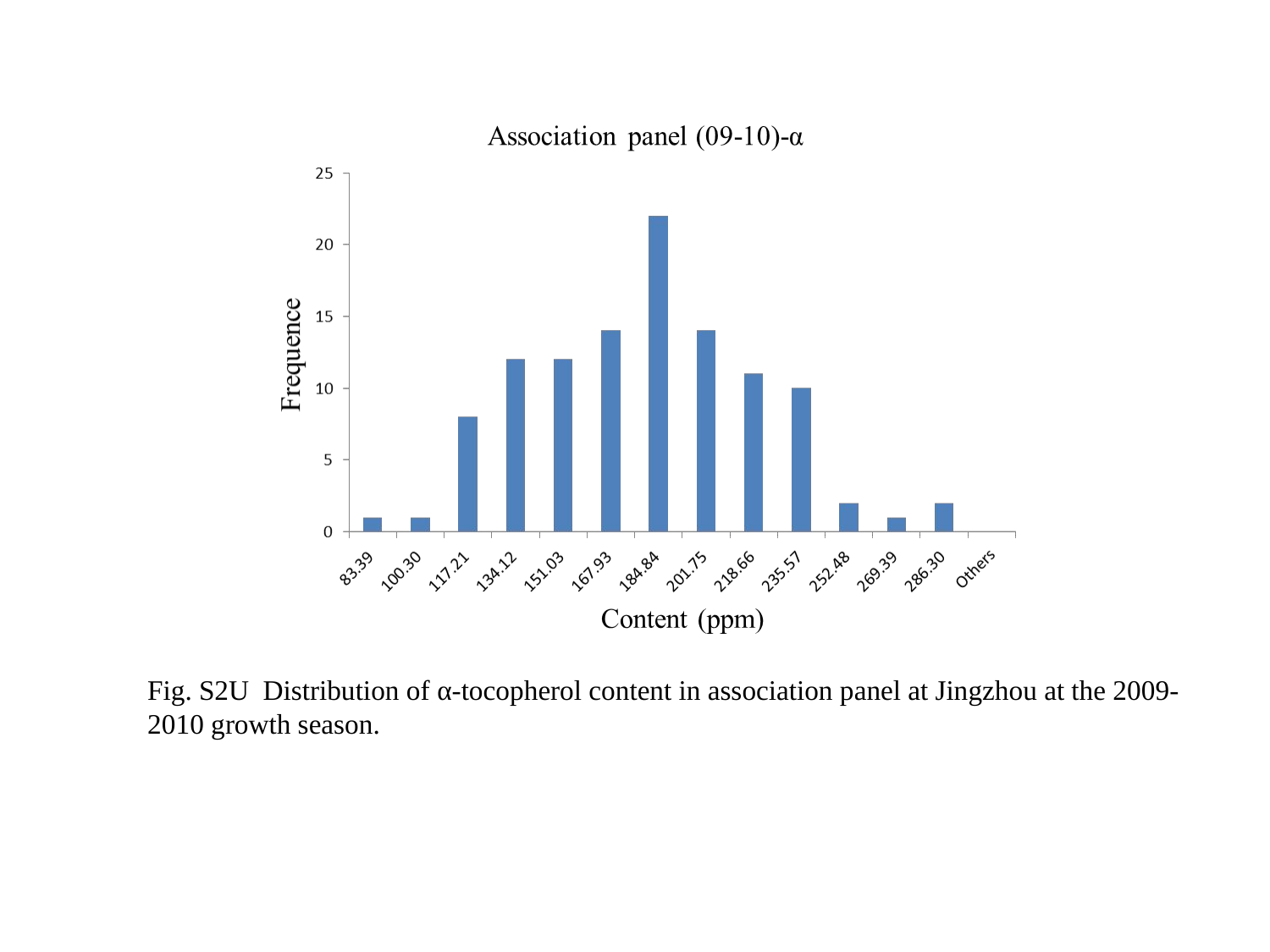

Fig. S2U Distribution of α-tocopherol content in association panel at Jingzhou at the 2009-2010 growth season.

## Slide 22
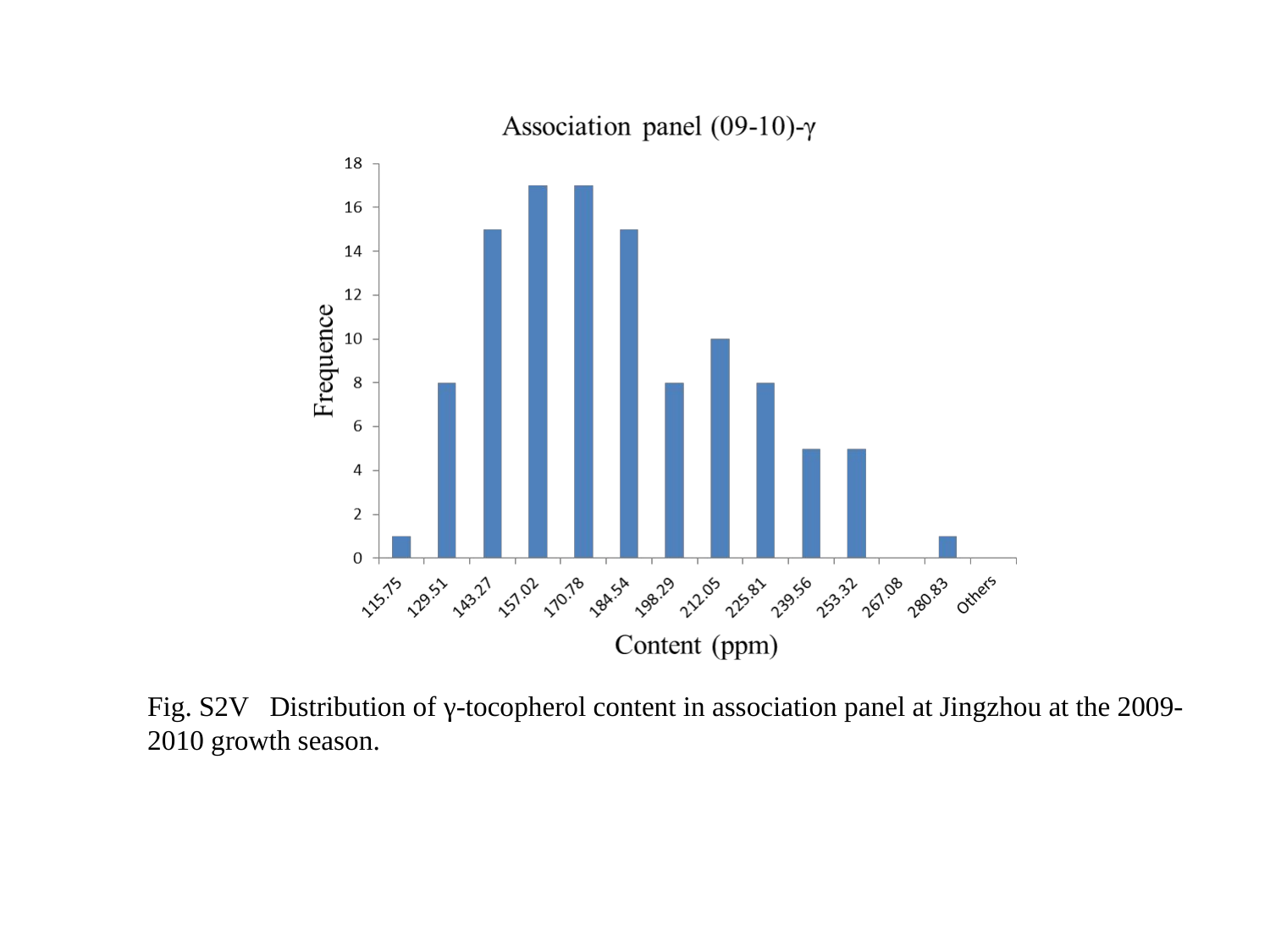

Fig. S2V Distribution of γ-tocopherol content in association panel at Jingzhou at the 2009-2010 growth season.

## Slide 23
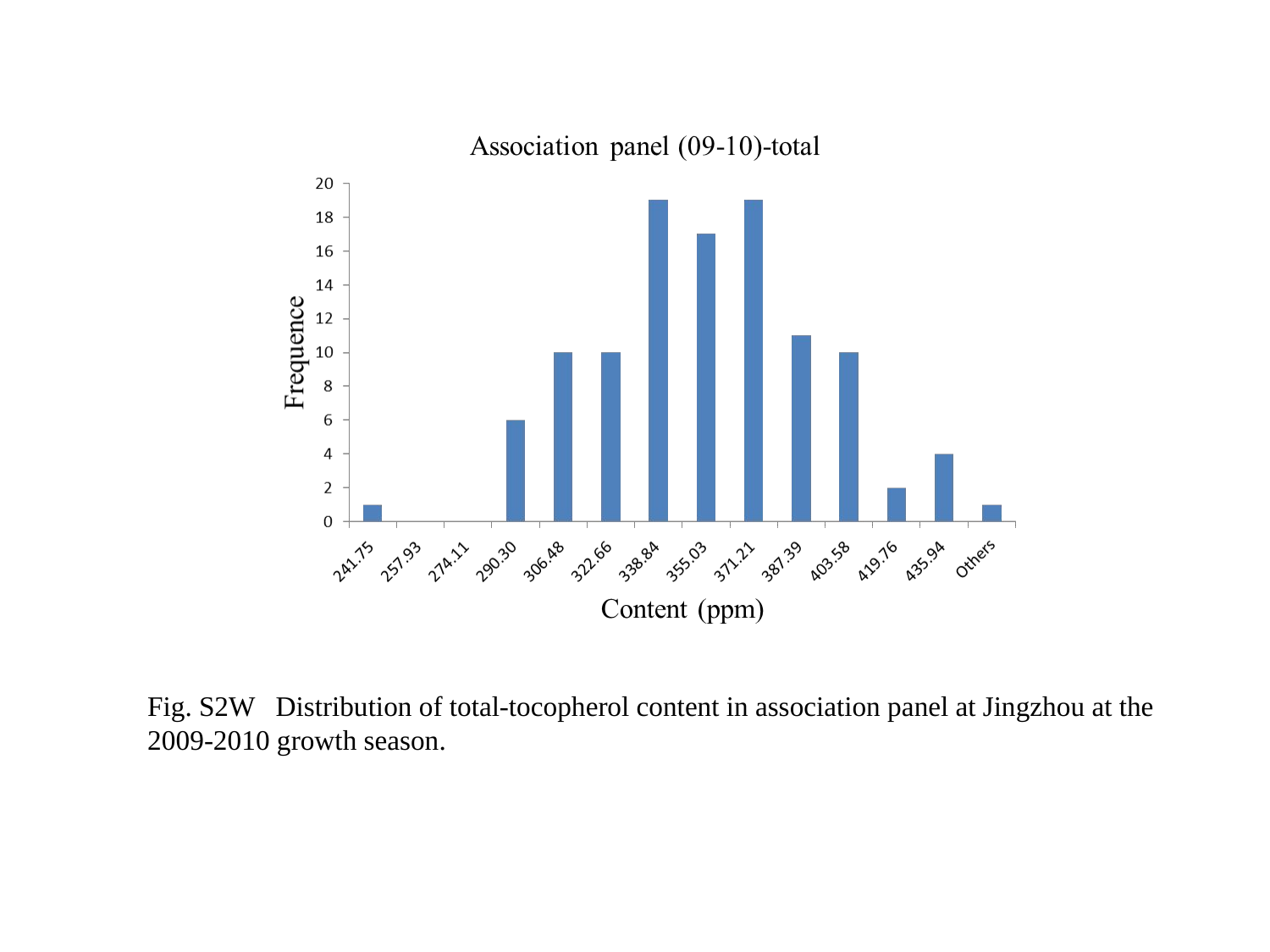

Fig. S2W Distribution of total-tocopherol content in association panel at Jingzhou at the 2009-2010 growth season.

## Slide 24
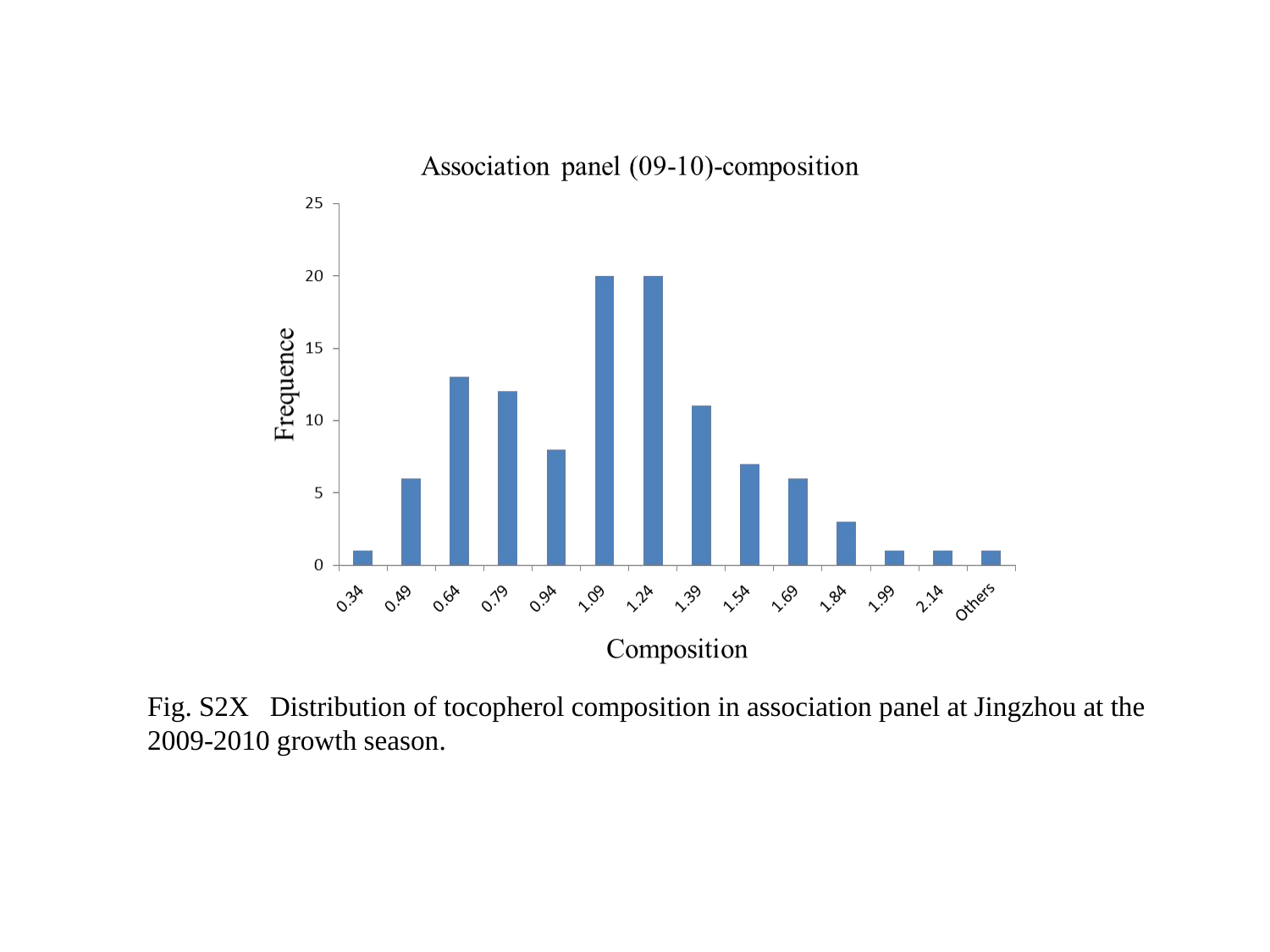

Fig. S2X Distribution of tocopherol composition in association panel at Jingzhou at the 2009-2010 growth season.
