# Supplementary material for: Unraveling the Genetic Basis of Seed Tocopherol Content and Composition in Rapeseed (Brassica napus L.)
Source: PLoS One. 2012 Nov 20;7(11):e50038. doi: 10.1371/journal.pone.0050038 (PMC3502226; doi:10.1371/journal.pone.0050038)
Supplement: Figure S4 — Schematic of the chromosomal components of the nearly isogenic lines by molecular markers in qTOC.A7 and qTOC.A9 . (PPT) [file pone.0050038.s004.ppt]

## Slide 1
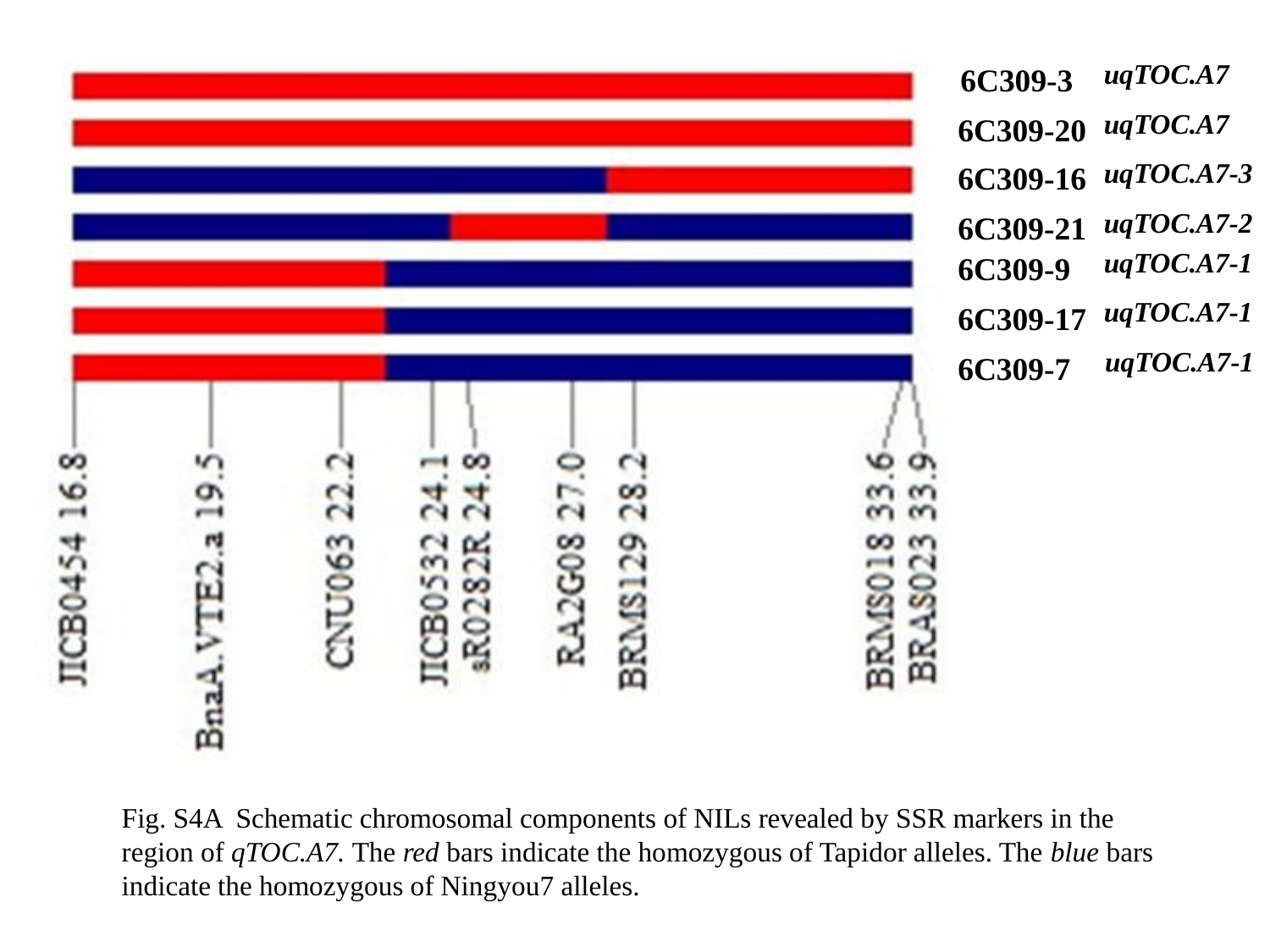

uqTOC.A7
uqTOC.A7
uqTOC.A7-3
uqTOC.A7-2
uqTOC.A7-1
uqTOC.A7-1
uqTOC.A7-1
6C309-3
6C309-20
6C309-16
6C309-21
6C309-9
6C309-17
6C309-7
Fig. S4A Schematic chromosomal components of NILs revealed by SSR markers in the region of qTOC.A7. The red bars indicate the homozygous of Tapidor alleles. The blue bars indicate the homozygous of Ningyou7 alleles.

## Slide 2
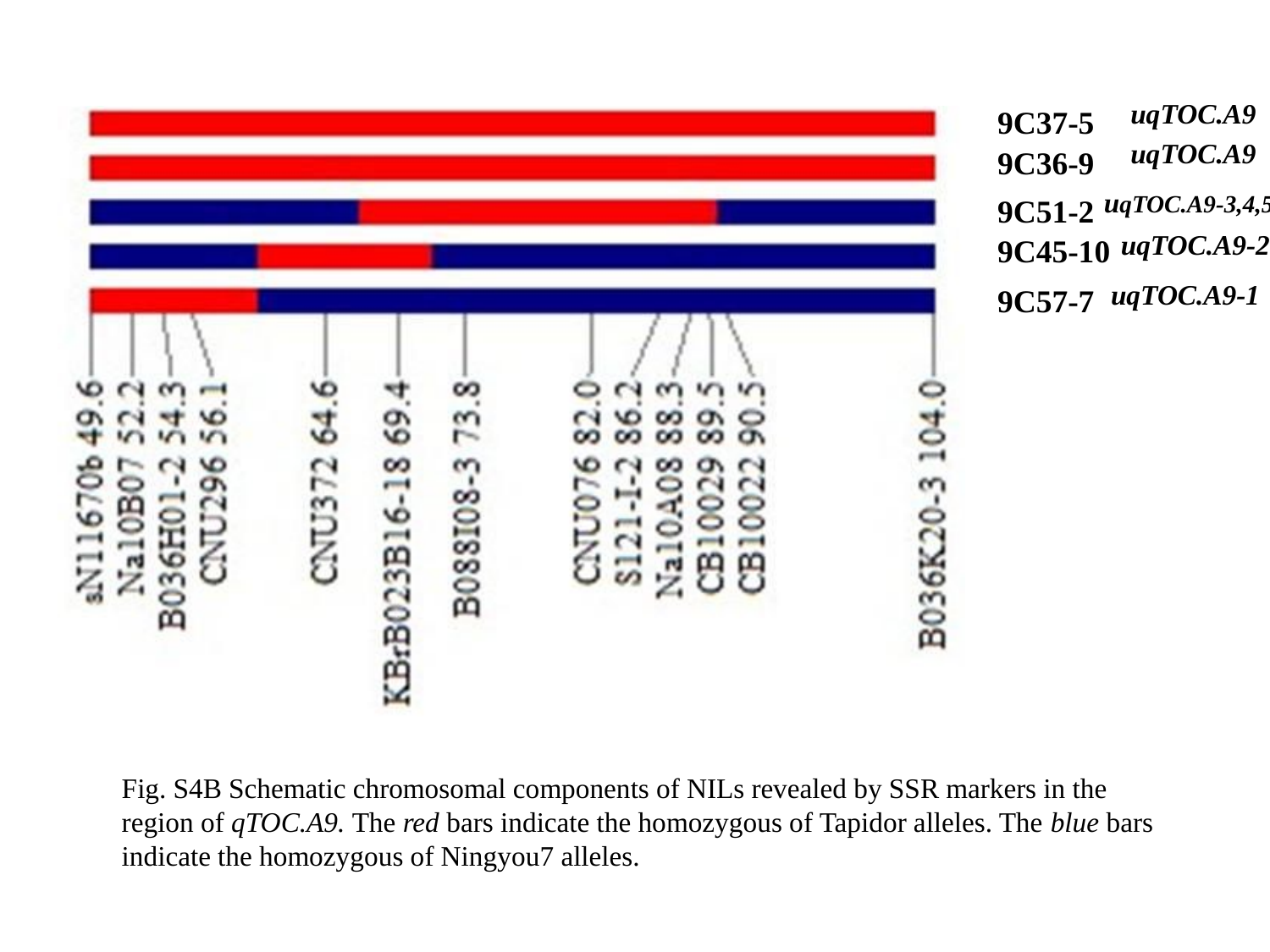

uqTOC.A9
 uqTOC.A9
uqTOC.A9-3,4,5
 uqTOC.A9-2
 uqTOC.A9-1
9C37-5
9C36-9
9C51-2
9C45-10
9C57-7
Fig. S4B Schematic chromosomal components of NILs revealed by SSR markers in the region of qTOC.A9. The red bars indicate the homozygous of Tapidor alleles. The blue bars indicate the homozygous of Ningyou7 alleles.
